# Supplementary material for: Functional divergence of CYP76AKs shapes the chemodiversity of abietane-type diterpenoids in genus Salvia
Source: Nat Commun. 2023 Aug 4;14:4696. doi: 10.1038/s41467-023-40401-y (PMC10403556; doi:10.1038/s41467-023-40401-y)
Supplement: Supplementary file 1 — Supplementary Information [file 41467_2023_40401_MOESM1_ESM.pdf]

# SUPPLEMENTARY INFORMATION

## Functional divergence of CYP76AKs shapes the chemodiversity of abietane-type diterpenoids in genus *Salvia*

Hu *et al.*

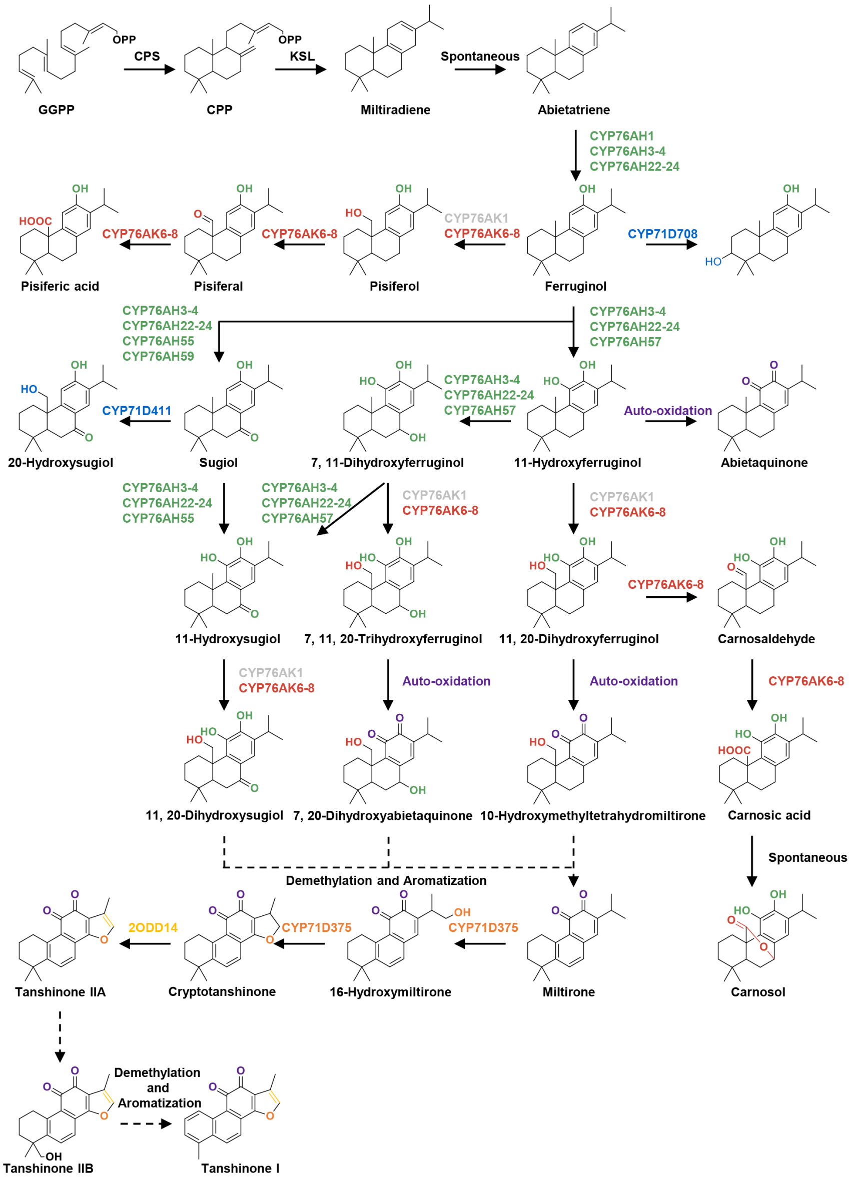


**Supplementary Figure 1. Proposed ATDs biosynthetic pathway in the genus *Salvia*[25-30].** Based on the biosynthesis study of ATDs in *Salvia* species (*S. pomifera*, *S. fruticosa*, *S. rosmarinus*, *S. officinalis*, and *S. miltiorrhiza*), miltiradiene is derived from GGPP and catalyzed by CPS and KSL terpene synthases, which spontaneously oxidizes to abietatriene. The CYP76AH1 enzyme (green) then oxidize abietatriene to the precursor ferruginol, while the 11-hydroxyferruginol synthase (CYP76AH3-4/22-24, green) further oxidizes ferruginol to 11-hydroxyferruginol, 7,11-dihydroxyferruginol, sugiol and 11-hydroxysugiol. In addition, CYP76AK1 (grey) catalyzes oxidation at C-20 to generate an alcohol group, while CYP76AK6-8 (red) carry out hydroxylation, carbonylation, and carboxylation at C-20 to oxidize ferruginol and 11-hydroxyferruginol to pisiferic acid and carnosic acid, respectively. However, the mechanism of coupled demethylation (C20) and aromatization (ring B) reactions to form miltirone, as well as the demethylation (C19) and aromatization of ring A, remain unclear. CYP71D375 (orange) is known to generate the 14,16-epoxy D-ring, which is then further oxidized to a furan ring by 2ODD14 (yellow) during tanshinones biosynthesis. The citation of the literatures is consistent with the manuscript.

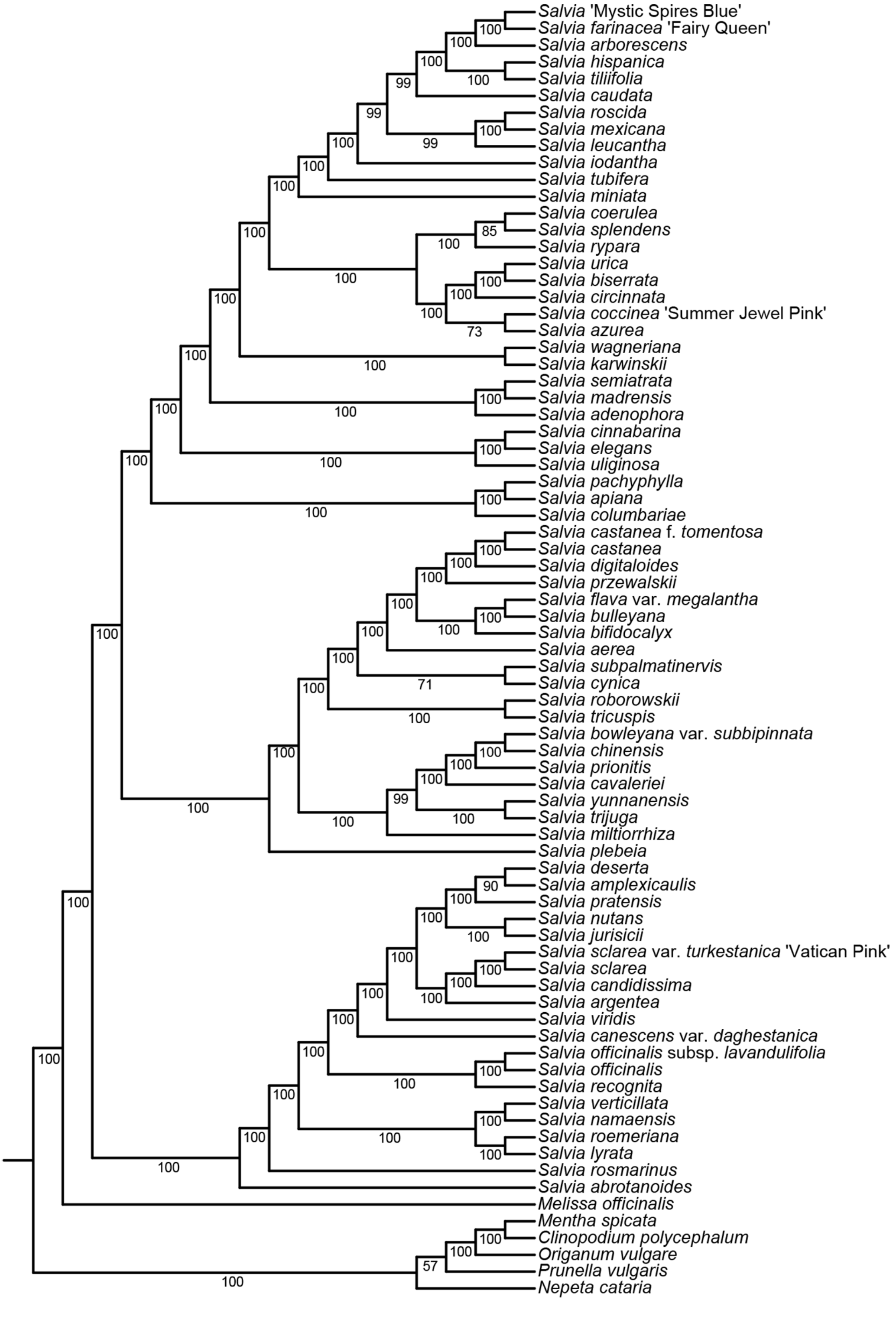


**Supplementary Figure 2. A phylogeny inferred from 2178 OGs by ASTRAL.**
Bootstrap values are presented near the nodes. Source data are provided as a Source Data file.

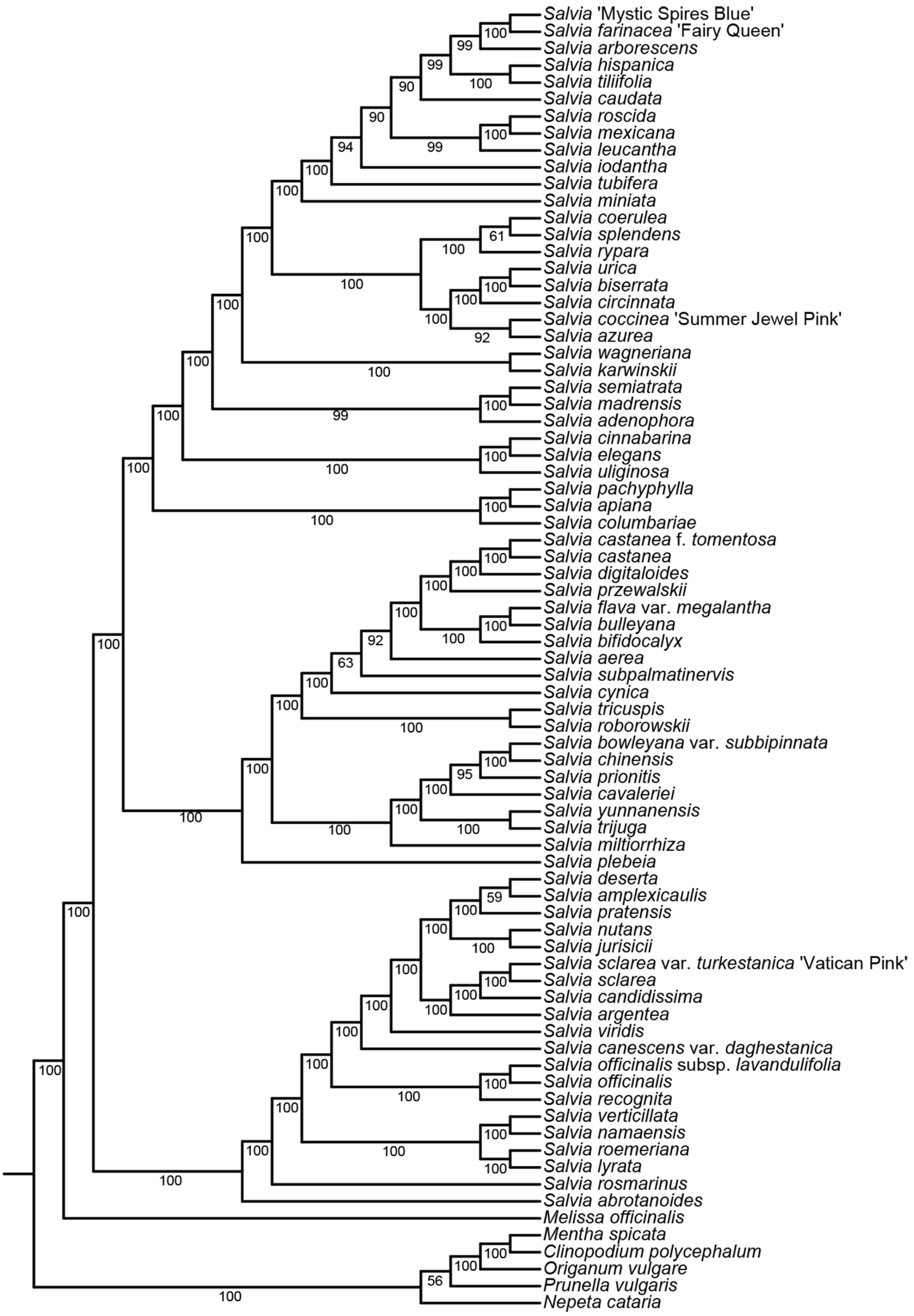


**Supplementary Figure 3. A phylogeny inferred from 1532 OGs by ASTRAL.**

Bootstrap values are presented near the nodes. Source data are provided as a Source Data file.

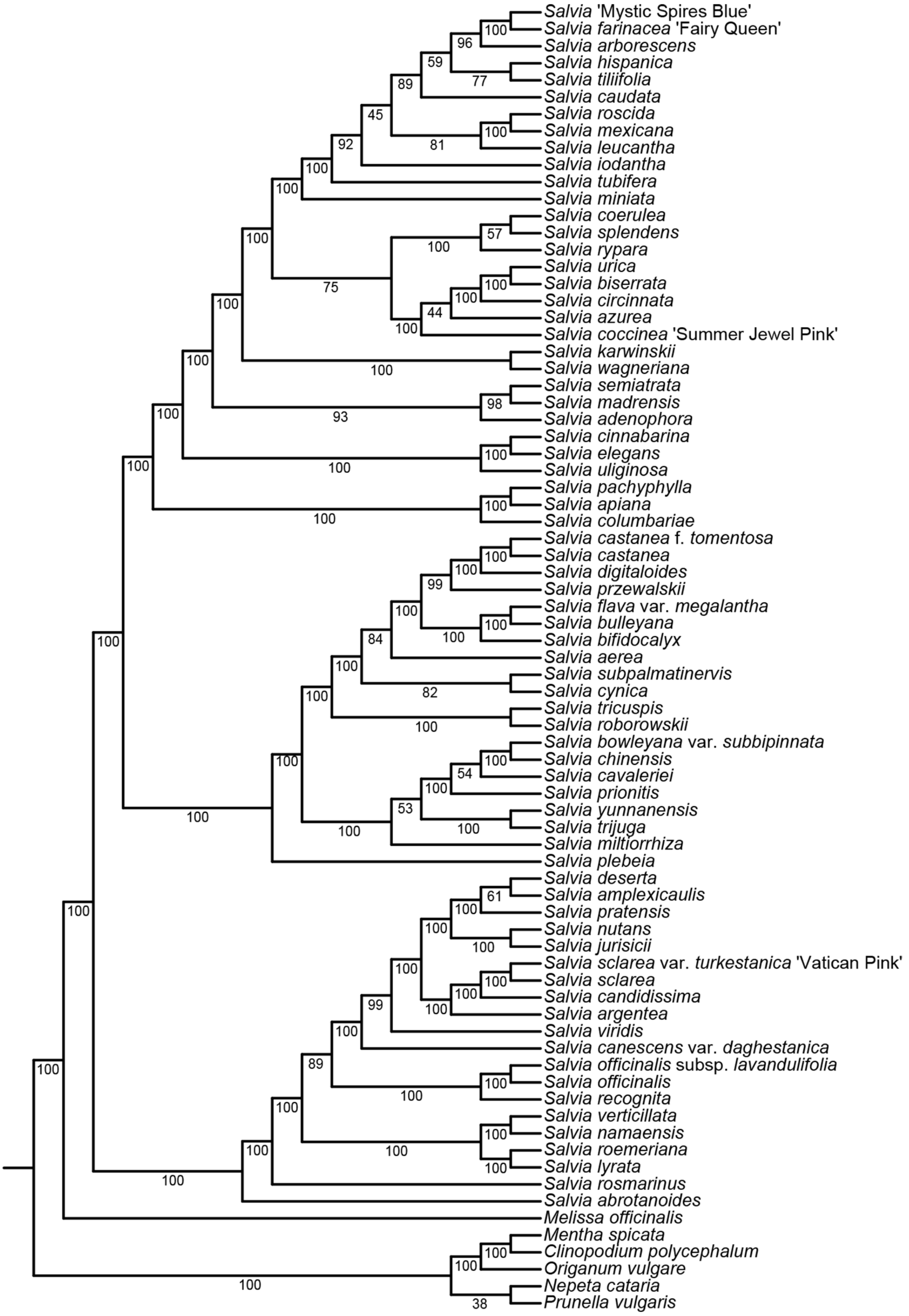


**Supplementary Figure 4. A phylogeny inferred from 1169 OGs by ASTRAL.**

Bootstrap values are presented near the nodes. Source data are provided as a Source Data file.

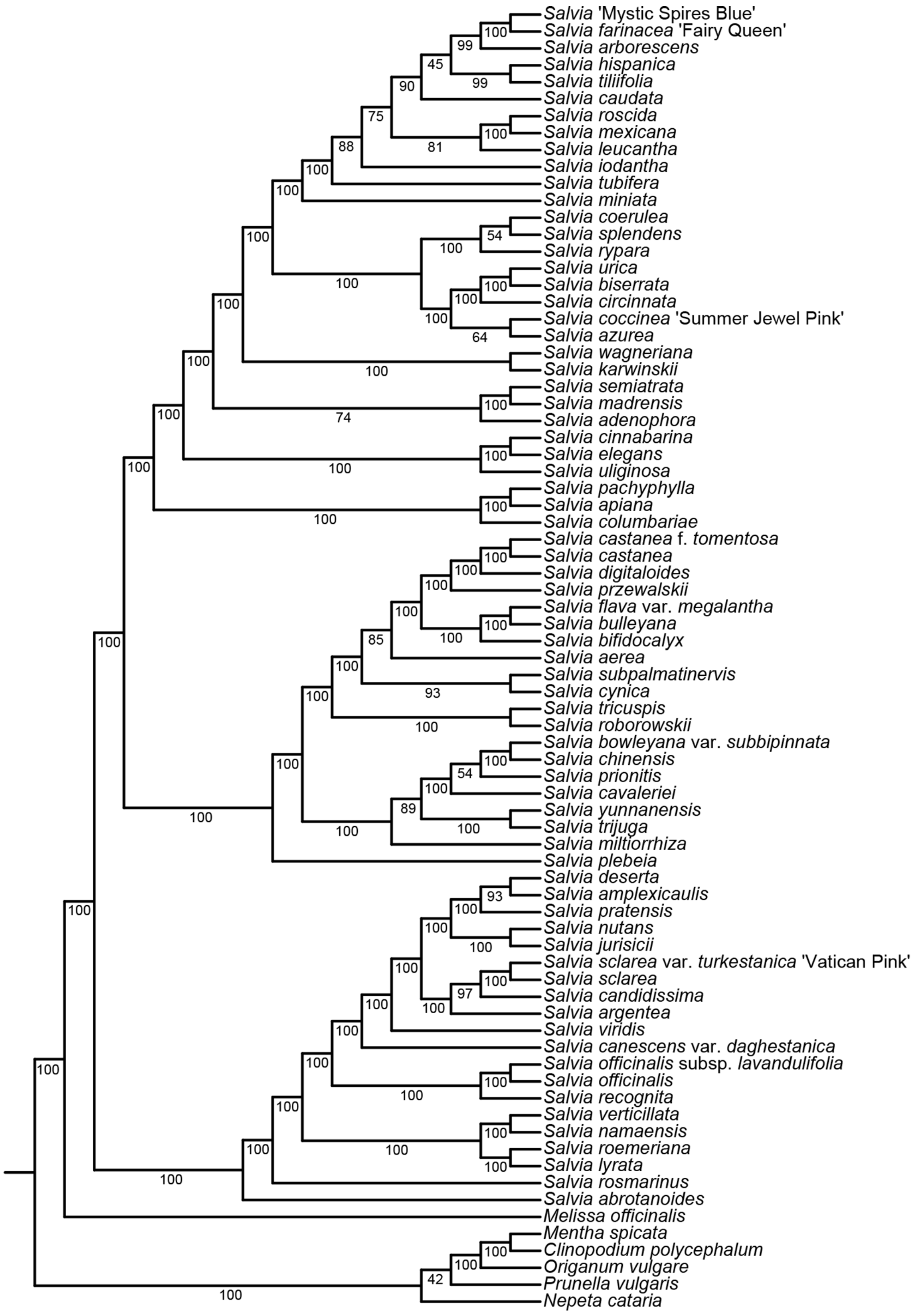


**Supplementary Figure 5. A phylogeny inferred from 512 OGs by ASTRAL.**

Bootstrap values are presented near the nodes. Source data are provided as a Source Data file.

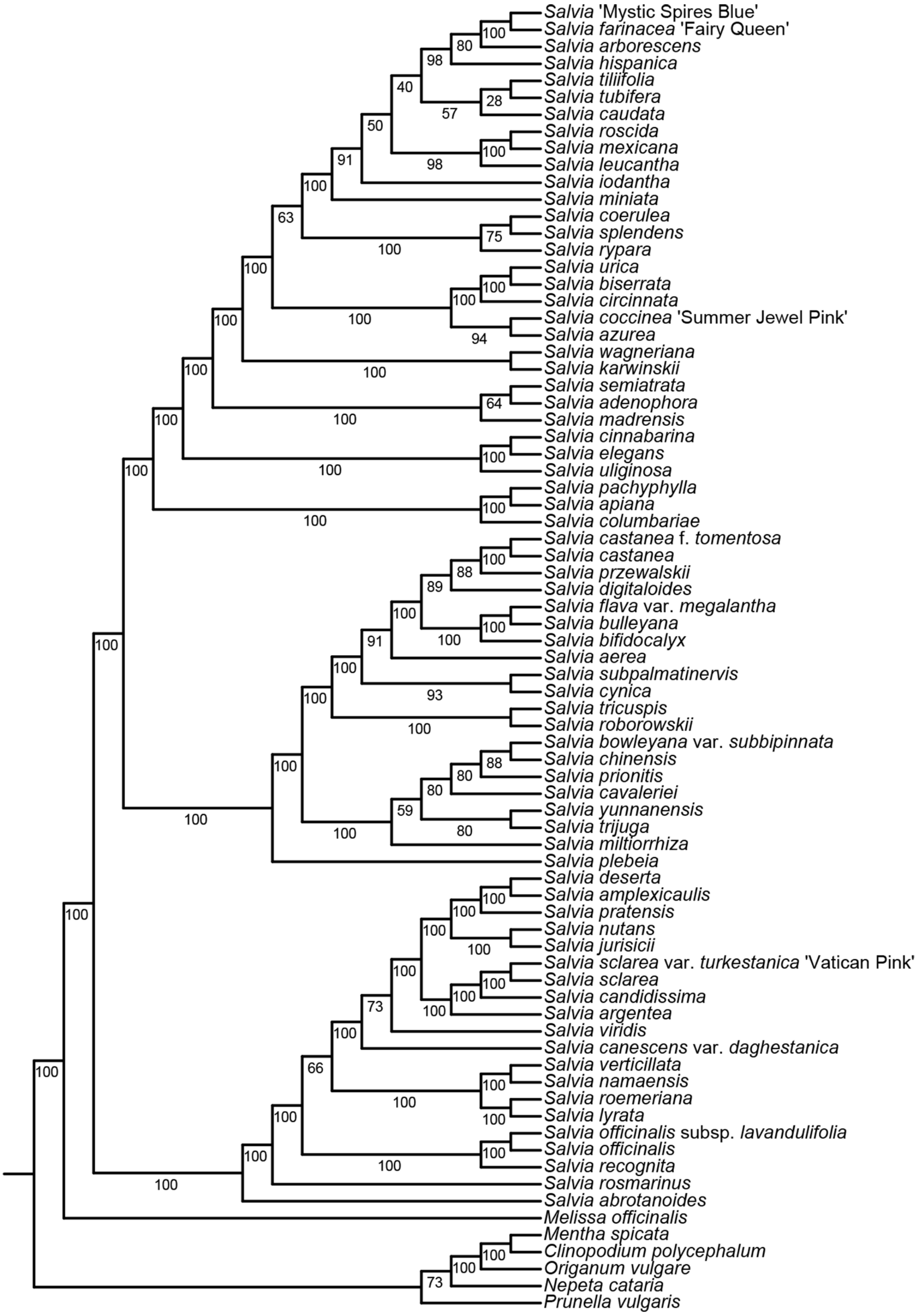


**Supplementary Figure 6. A phylogeny inferred from 130 OGs by ASTRAL.**

Bootstrap values are presented near the nodes. Source data are provided as a Source Data file.

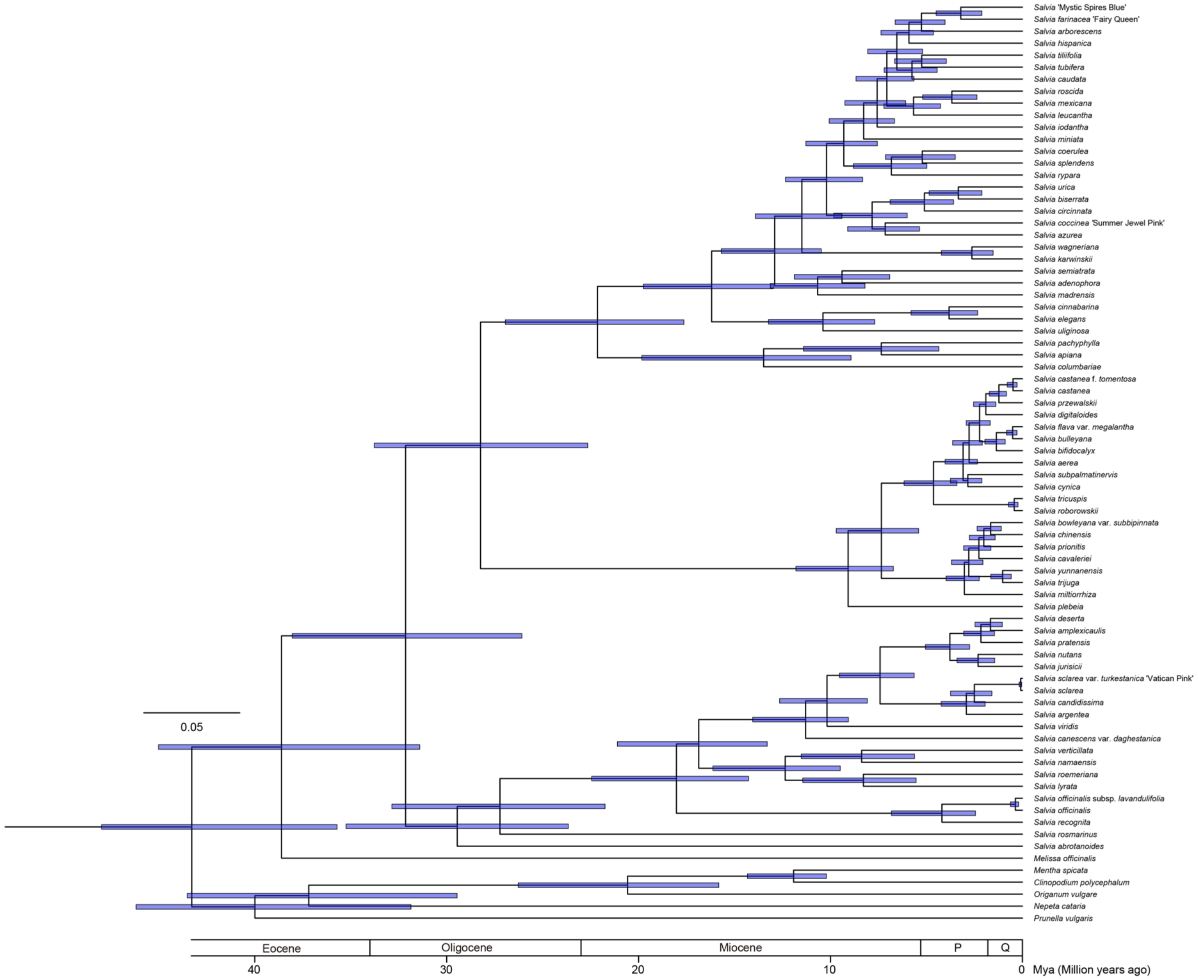


**Supplementary Figure 7. Time tree of *Salvia*.** The blue bars represent the range of 95% credibility intervals. Ages correspond to the geological timescale at bottom. Bar represented 0.05 Mya. Source data are provided as a Source Data file.

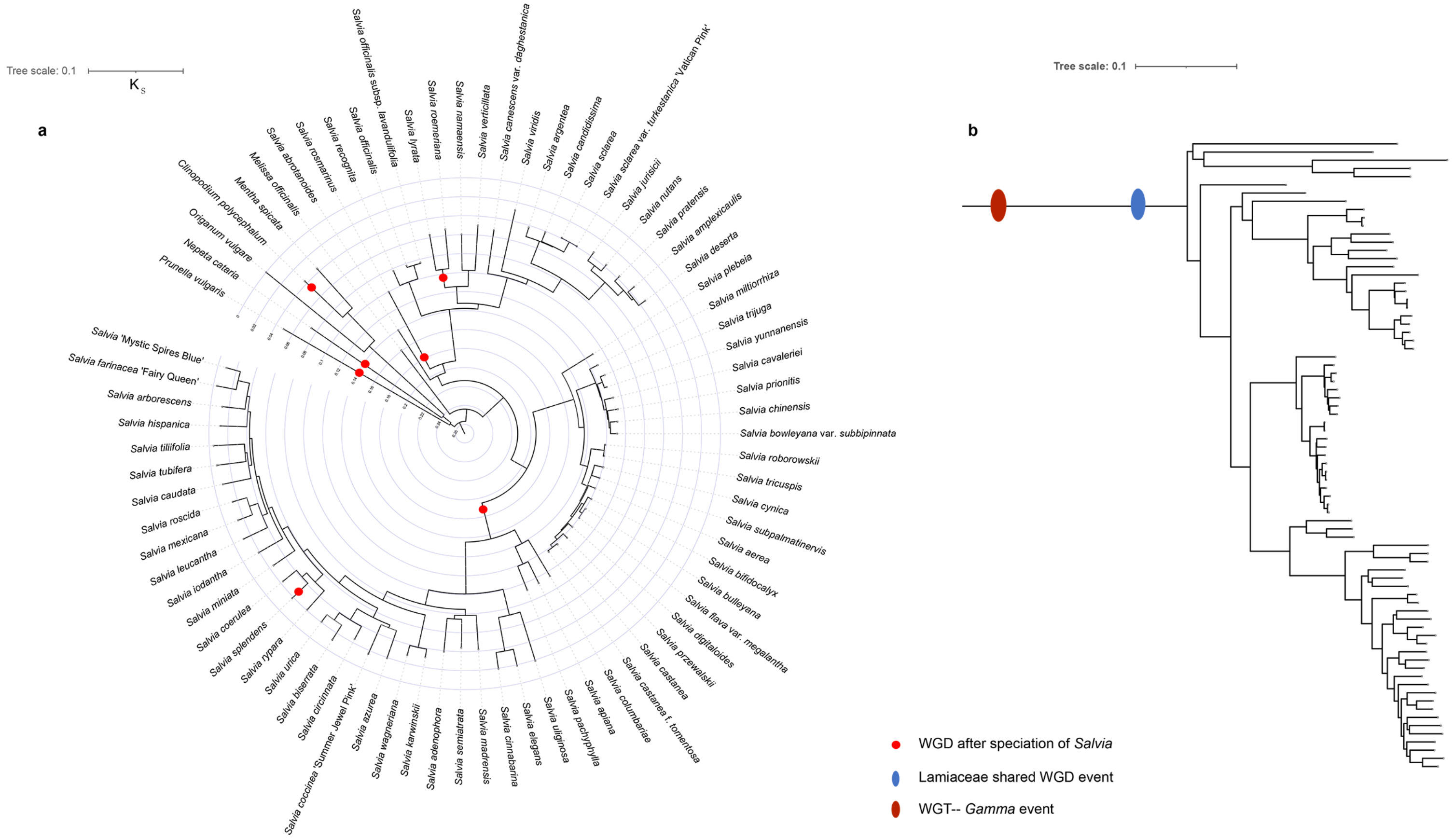


**Supplementary Figure 8. Whole-genome duplication (WGD) events of all species.** (a) Distribution of WGD events among *Salvia*. The branch length was represented in $K_S$ units. Red dots denote the putative WGD events that occurred after speciation of *Salvia*. (b) The WGD event that is common for plants or Lamiaceae. The red oval denotes WGT-- *Gamma* event. The blue oval denotes Laminaceae shared WGD event. Source data are provided as a Source Data file.

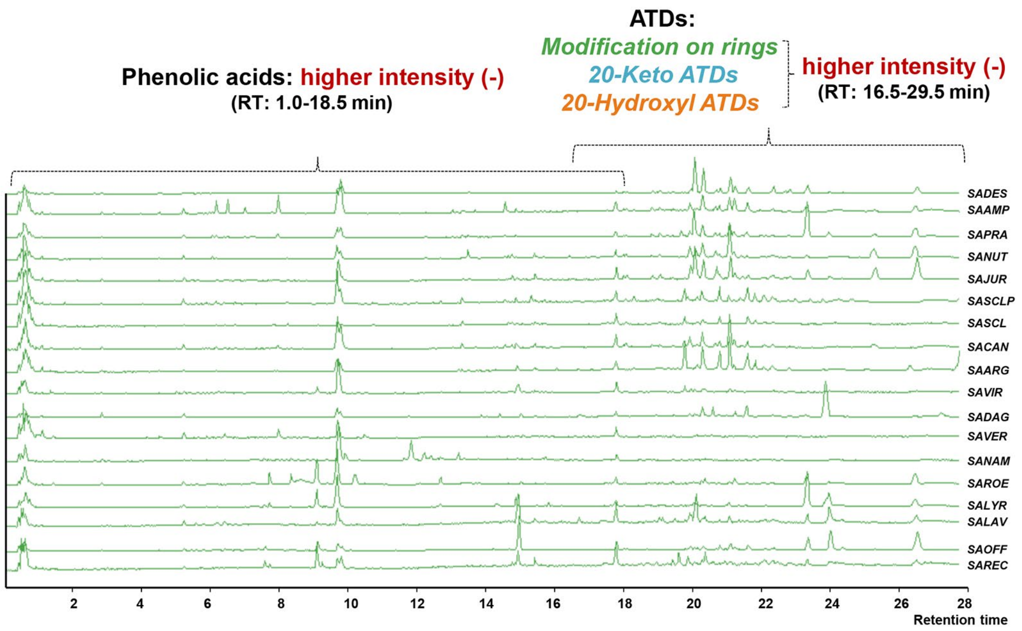


**Supplementary Figure 9. Total ion current (TIC) chromatograms of the 18 root samples in Clade I in the negative-ion mode.**

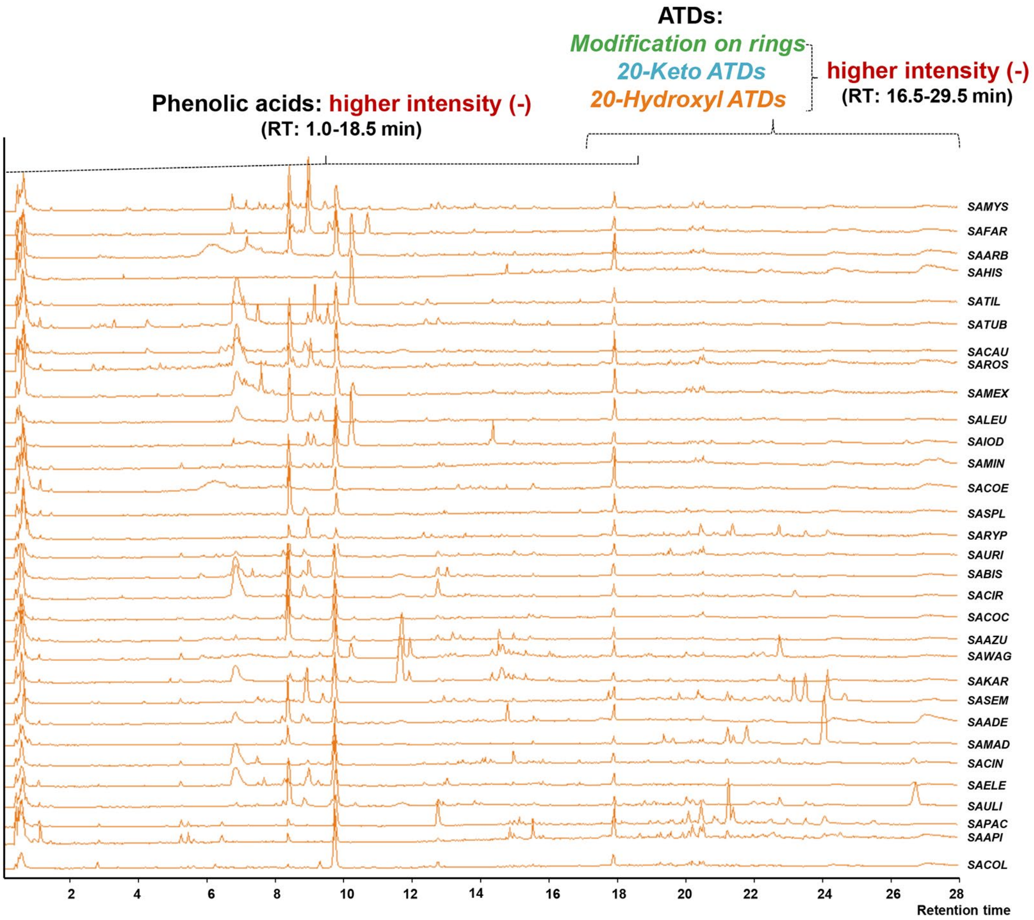


**Supplementary Figure 10. Total ion current (TIC) chromatograms of the 31 root samples in Clade II in the negative-ion mode.**

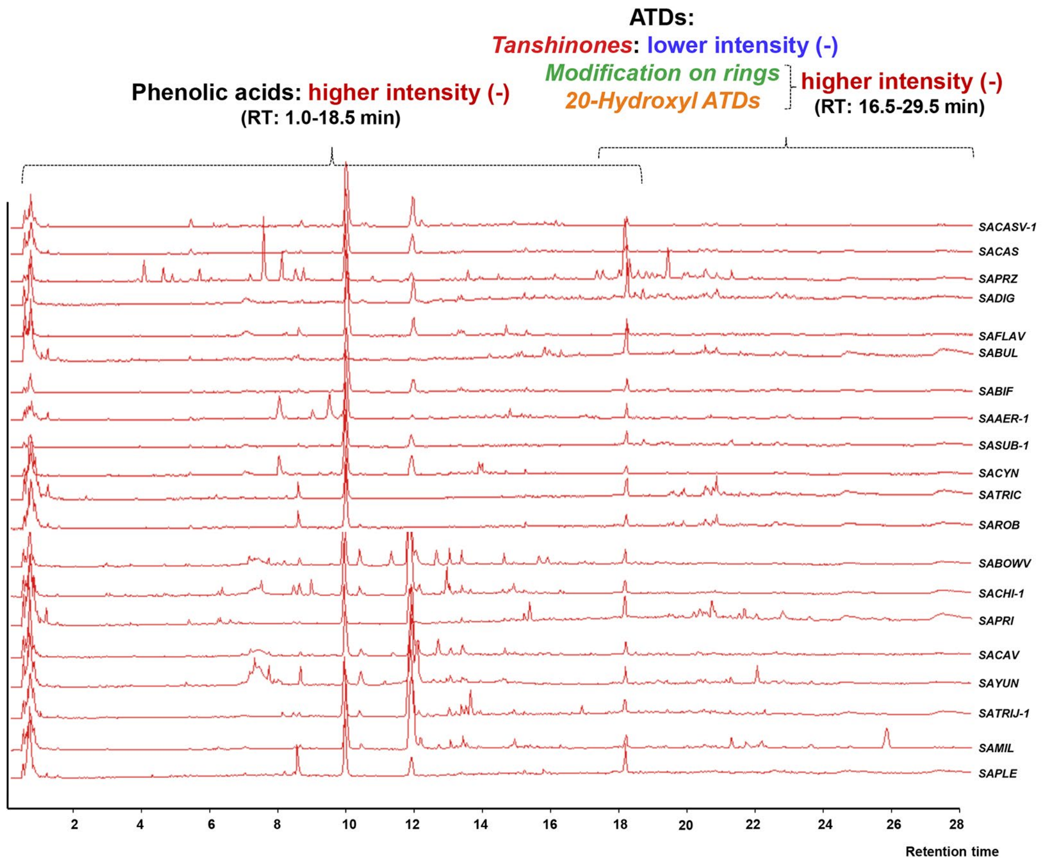


**Supplementary Figure 11. Total ion current (TIC) chromatograms of the 20 root samples in Clade IV in the negative-ion mode.**

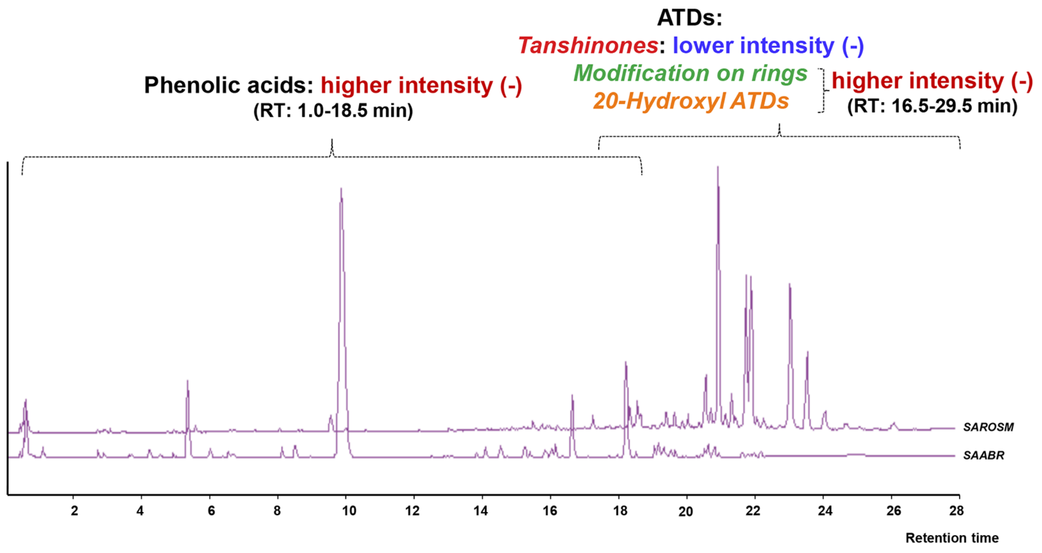


**Supplementary Figure 12. Total ion current (TIC) chromatograms of the 2 root samples in the subgenera *Perovskia* and *Rosmarinus* in the negative-ion mode.**

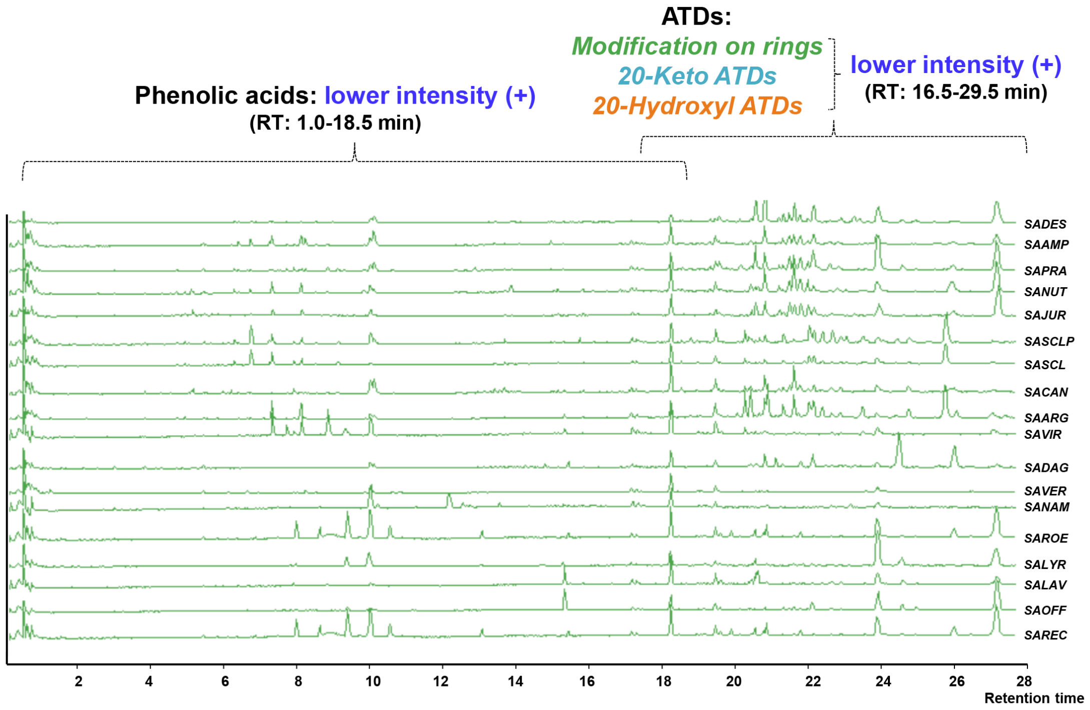


**Supplementary Figure 13. Total ion current (TIC) chromatograms of the 18 root samples in Clade I in the positive-ion mode.**

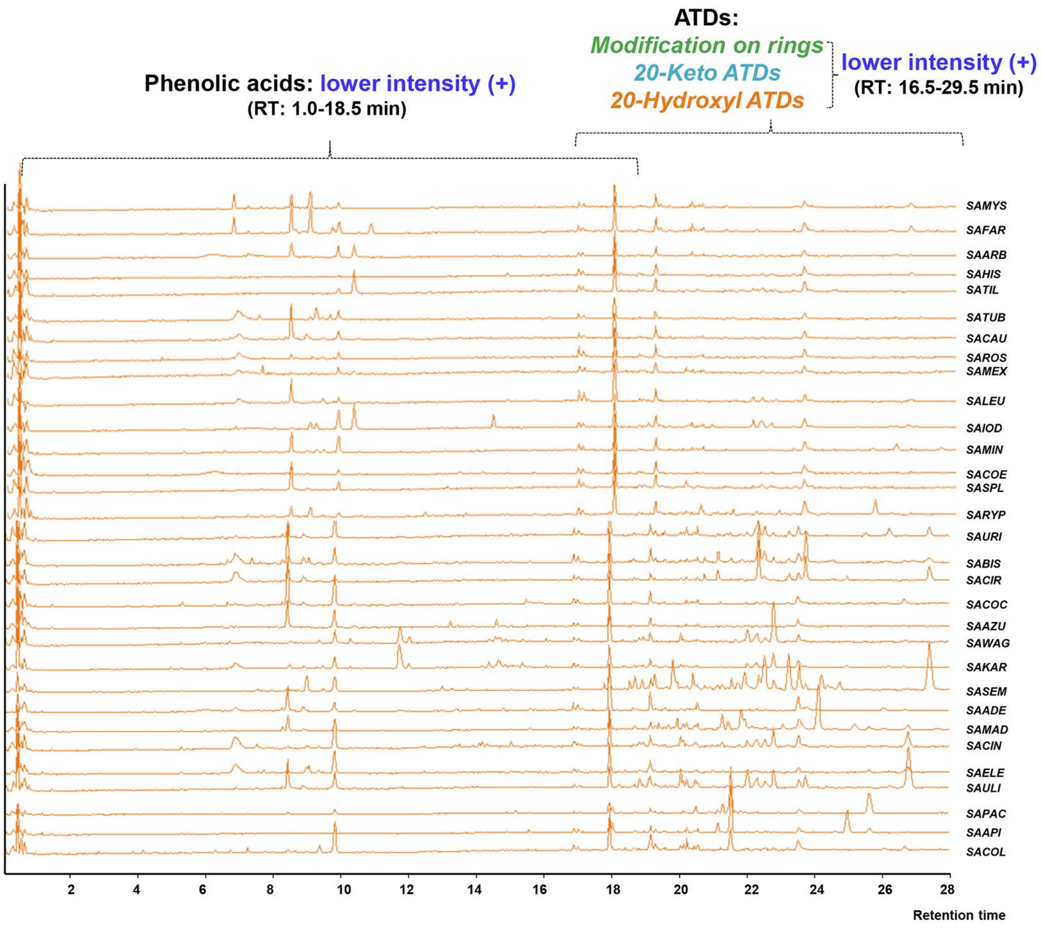


**Supplementary Figure 14. Total ion current (TIC) chromatograms of the 31 root samples in Clade II in the positive-ion mode.**

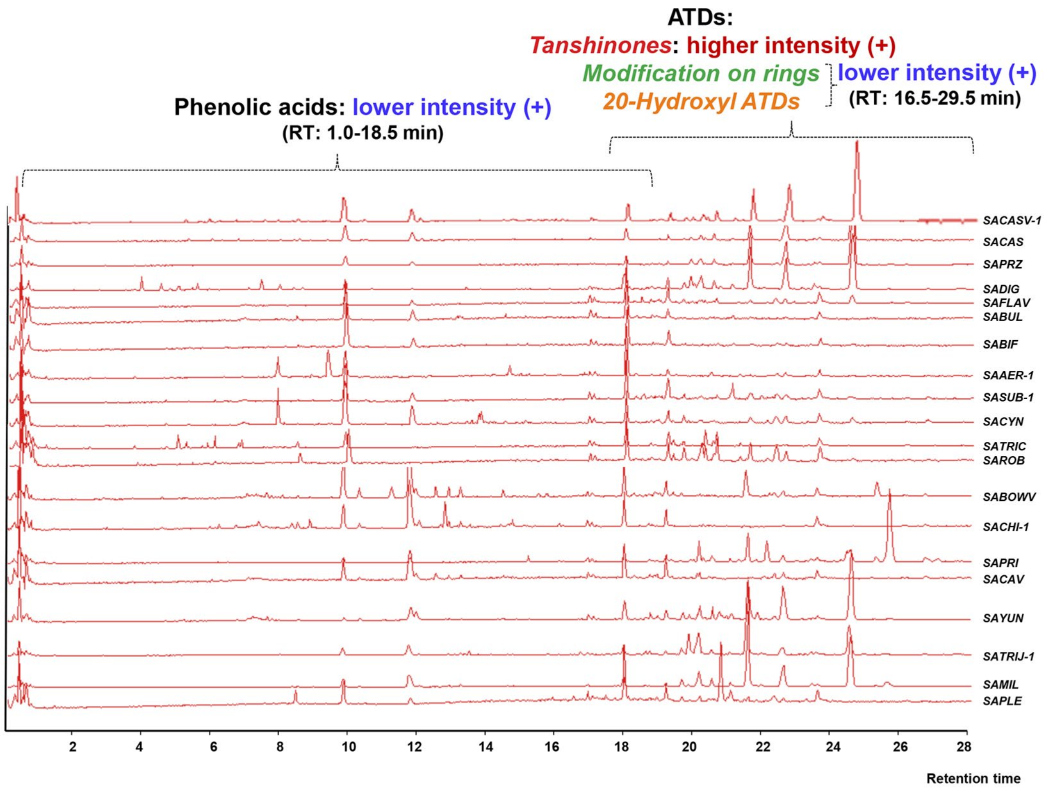


**Supplementary Figure 15. Total ion current (TIC) chromatograms of the 20 root samples in Clade IV in the positive-ion mode.**

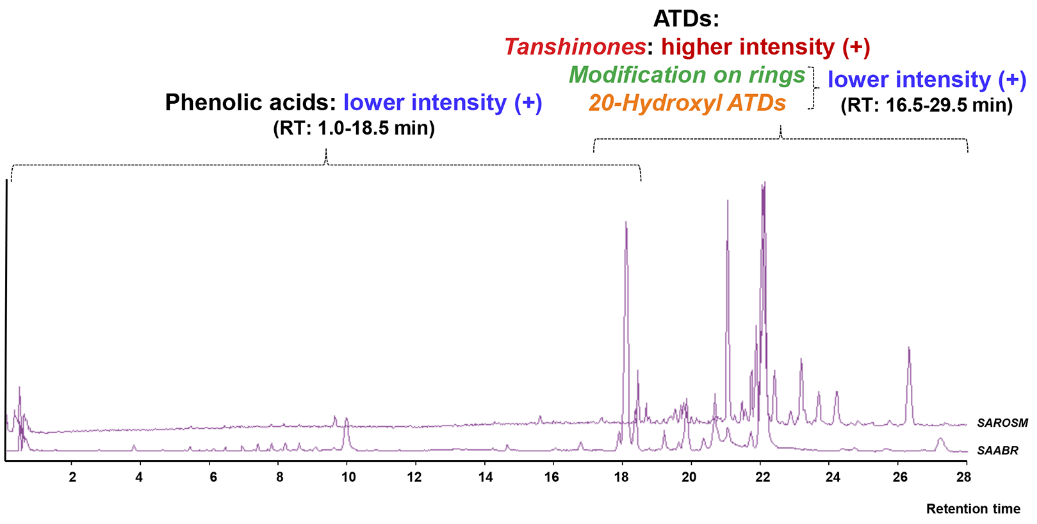


**Supplementary Figure 16. Total ion current (TIC) chromatograms of the 2 root samples in the subgenera *Perovskia* and *Rosmarinus* in the positive-ion mode.**

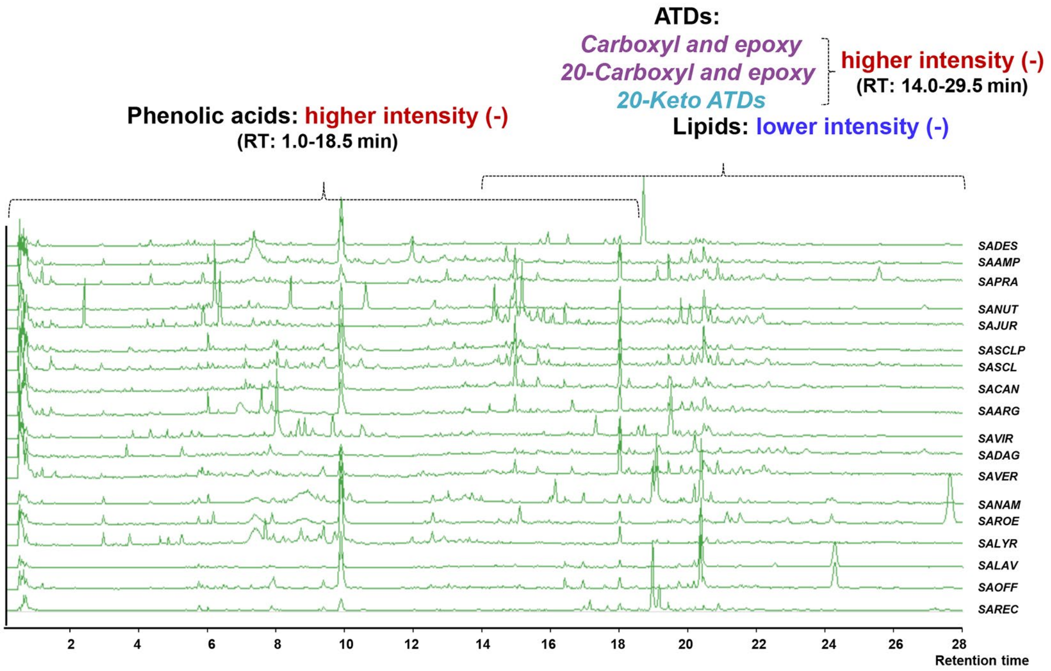


**Supplementary Figure 17. Total ion current (TIC) chromatograms of the 18 leaf samples in Clade I in the negative-ion mode.**

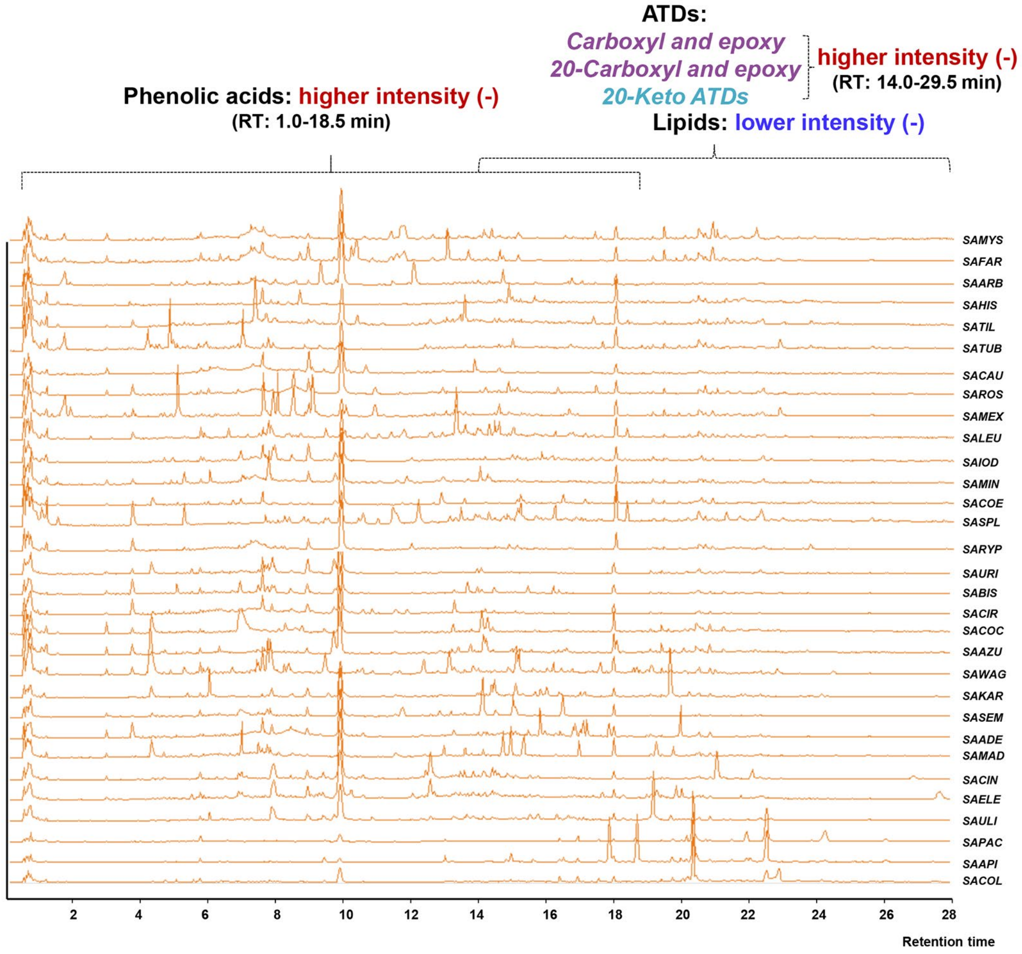


**Supplementary Figure 18. Total ion current (TIC) chromatograms of the 31 leaf samples in Clade II in the negative-ion mode.**

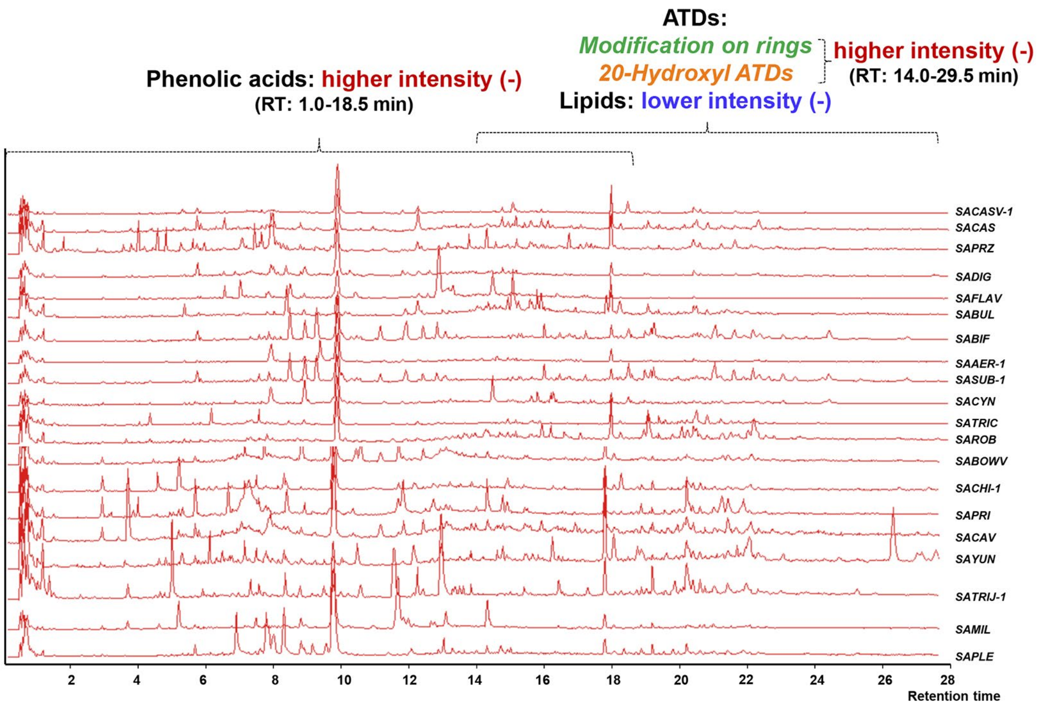


**Supplementary Figure 19. Total ion current (TIC) chromatograms of the 20 leaf samples in Clade IV in the negative-ion mode.**

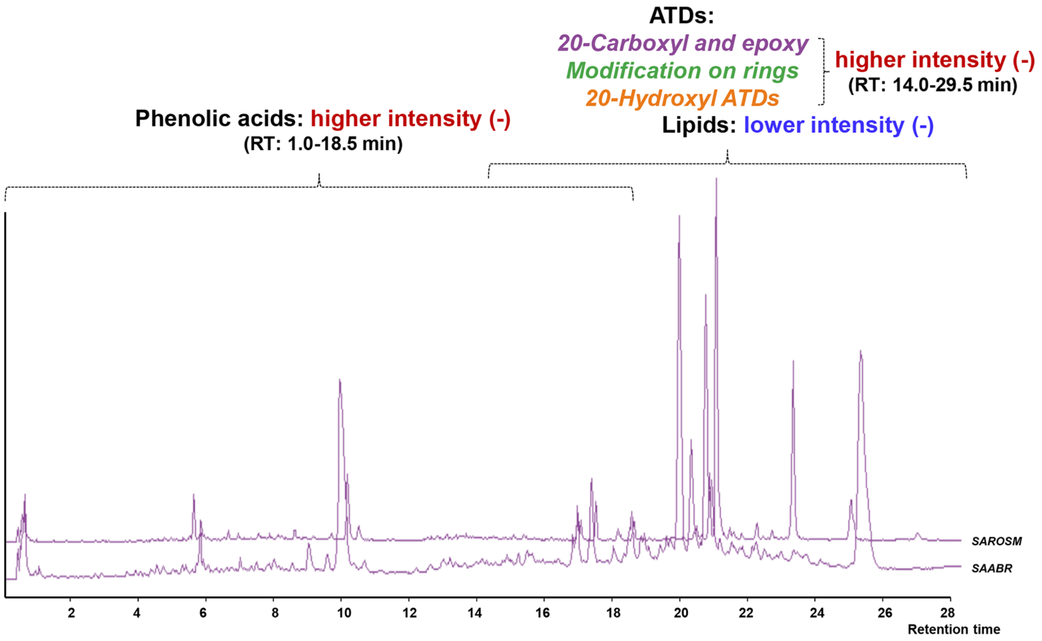


**Supplementary Figure 20. Total ion current (TIC) chromatograms of the 2 leaf samples in the subgenera *Perovskia* and *Rosmarinus* in the negative-ion mode.**

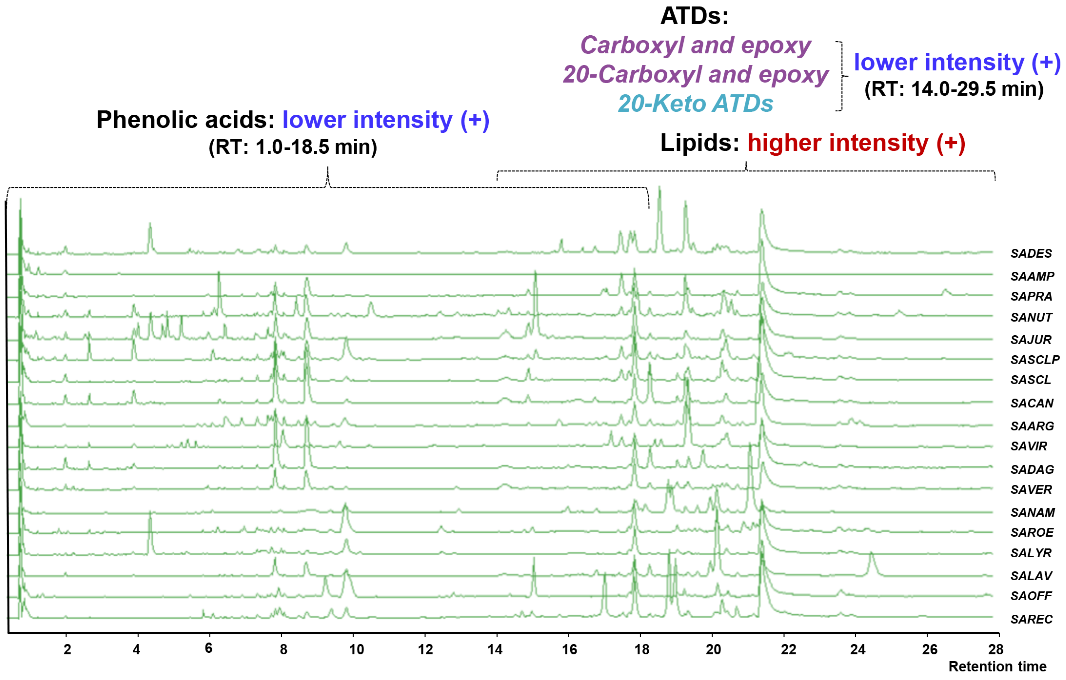


**Supplementary Figure 21. Total ion current (TIC) chromatograms of the 18 leaf samples in Clade I in the positive-ion mode.**

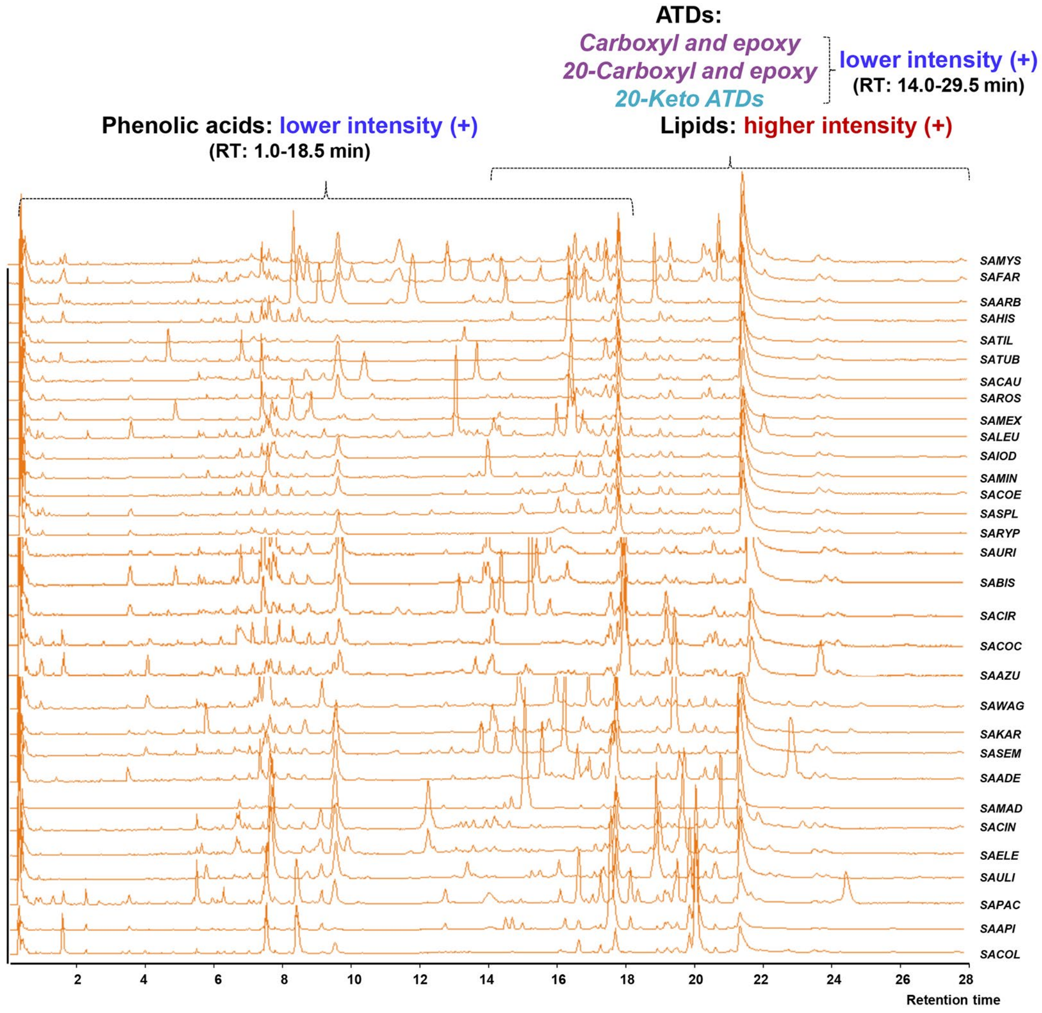


**Supplementary Figure 22. Total ion current (TIC) chromatograms of the 31 leaf samples in Clade II in the negative-ion mode.**

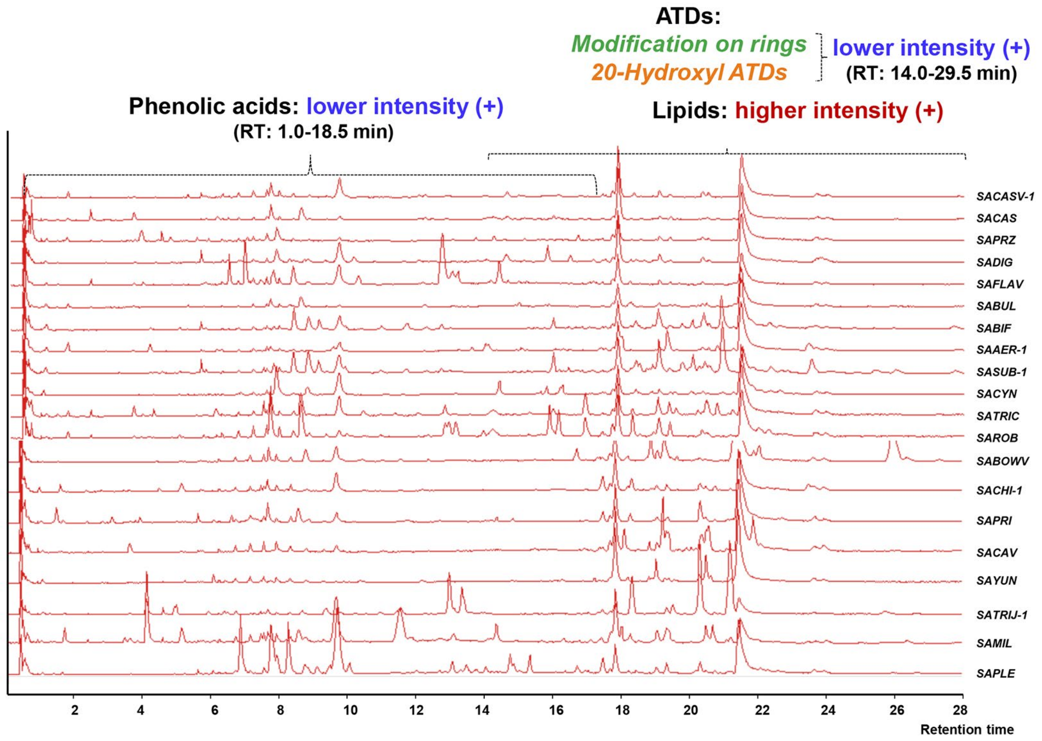


**Supplementary Figure 23. Total ion current (TIC) chromatograms of the 20 leaf samples in Clade IV in the positive-ion mode.**

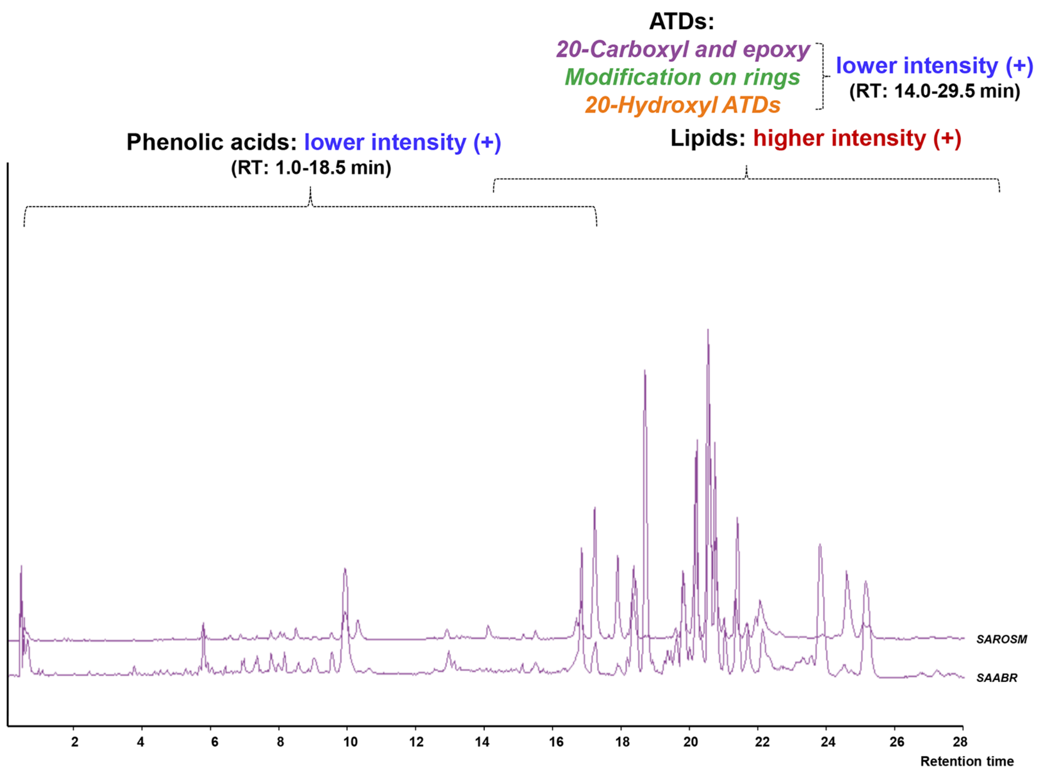


**Supplementary Figure 24. Total ion current (TIC) chromatograms of the 2 leaf samples in the subgenera *Perovskia* and *Rosmarinus* in the positive-ion mode.**

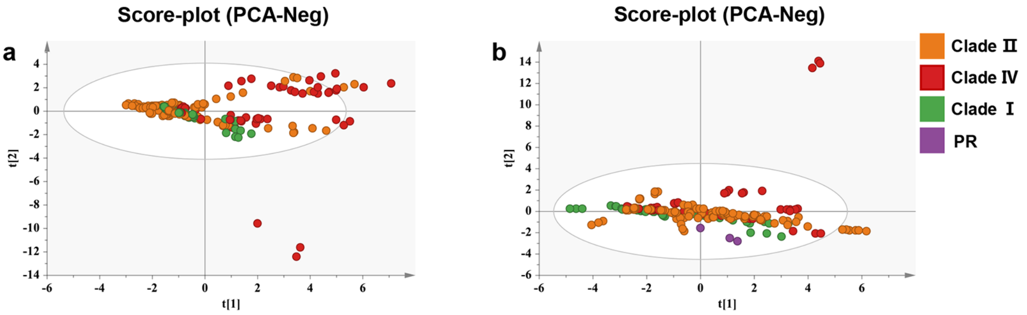


**Supplementary Figure 25. PCA analysis of 71 *Salvia* species based on the metabolites at retention time from 1.0 to 18.5 min in the negative-ion mode (phenolic acid fraction).** (a): Root samples; (b): leaf samples. PR, subgenera *Perovskia* and *Rosmarinus*

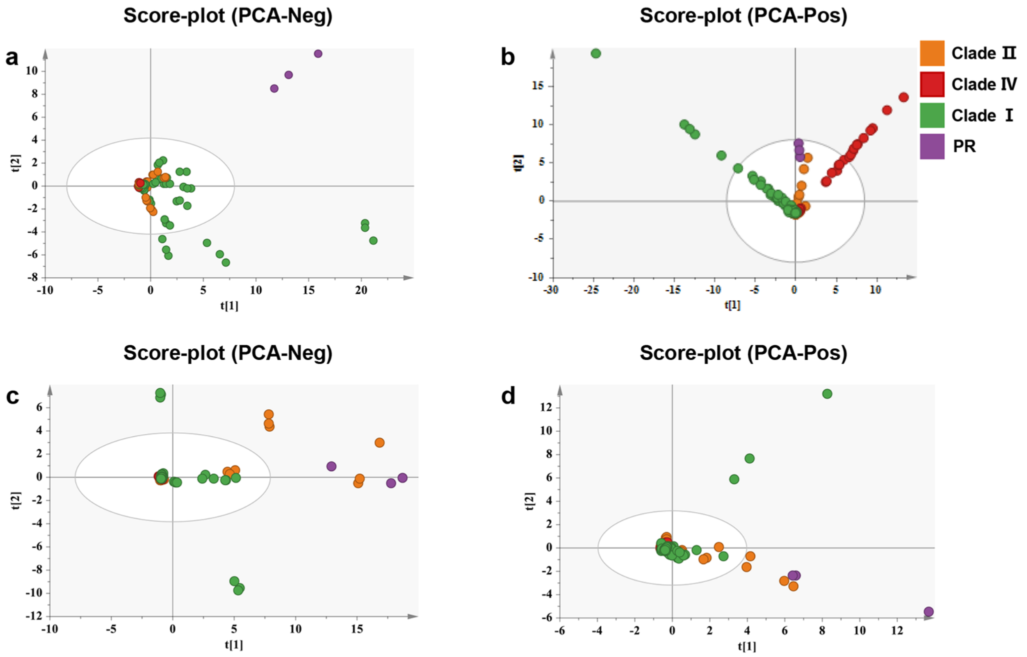


**Supplementary Figure 26. PCA analysis of 71 *Salvia* species based on the accumulation of ATDs (ATDs fraction).** (a). Root samples in the negative-ion mode (RT: 16.5-29.5 min); (b). Root samples in the positive-ion mode (RT: 16.5-29.5 min); (c) Leaf samples in the negative-ion mode (RT: 14.0-29.5 min); (d) Leaf samples in the positive-ion mode (RT: 14.0-29.5 min). PR, subgenera *Perovskia* and *Rosmarinus*

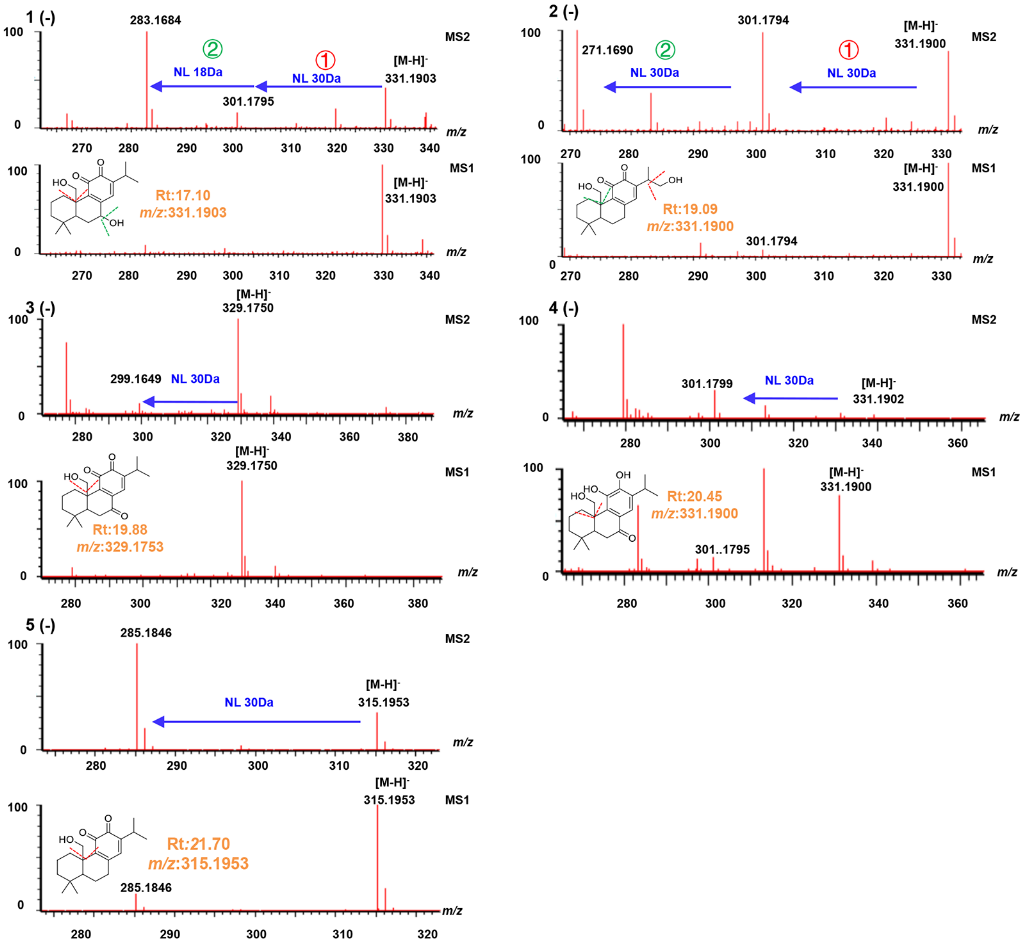


**Supplementary Figure 27. The fragmentation pattern of C-20 hydroxyl ATDs (Group-A) in the negative-ion mode.**

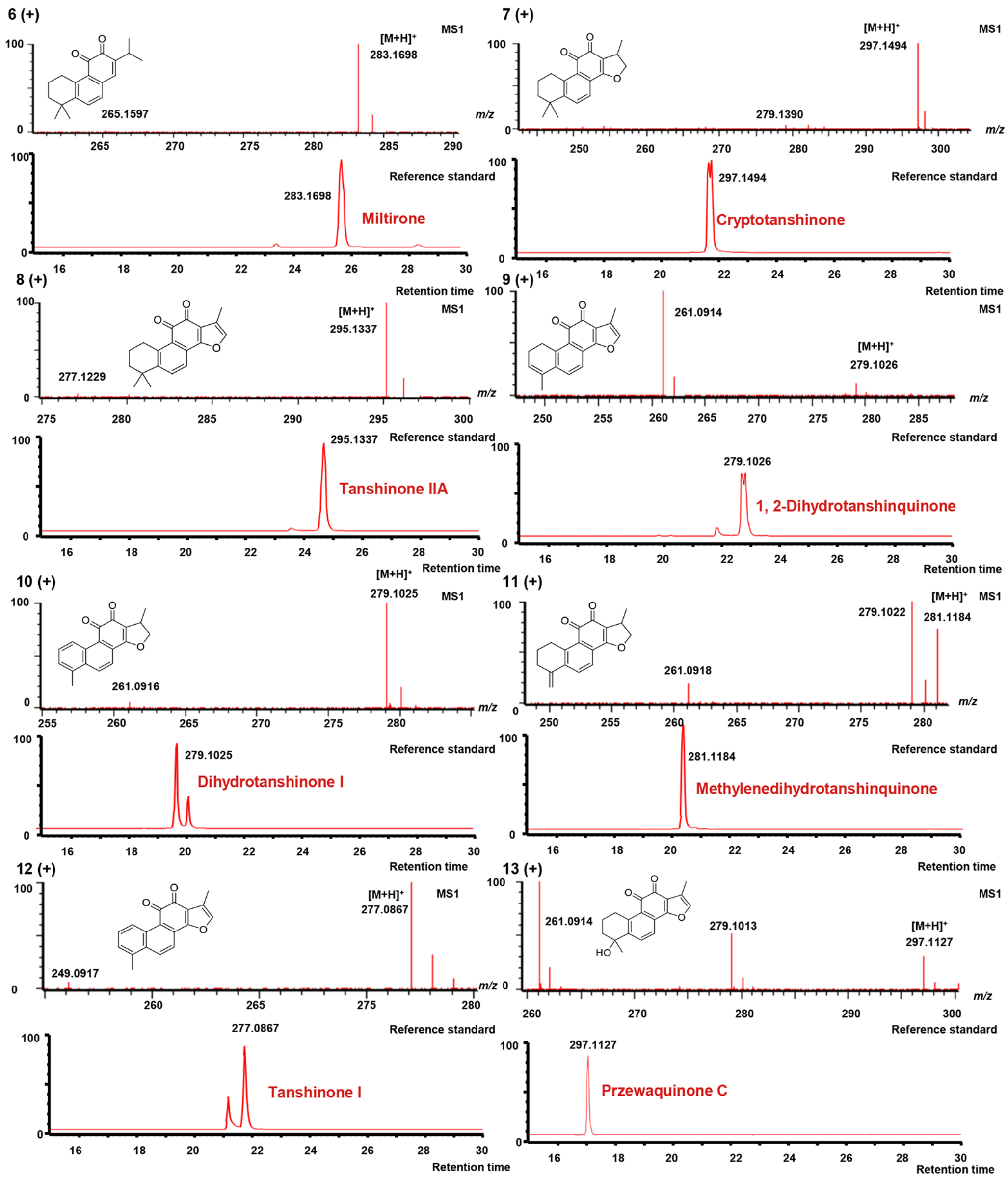


**Supplementary Figure 28. The identification results of tanshinones (Group-B) based on the reference standards in the positive-ion mode.**

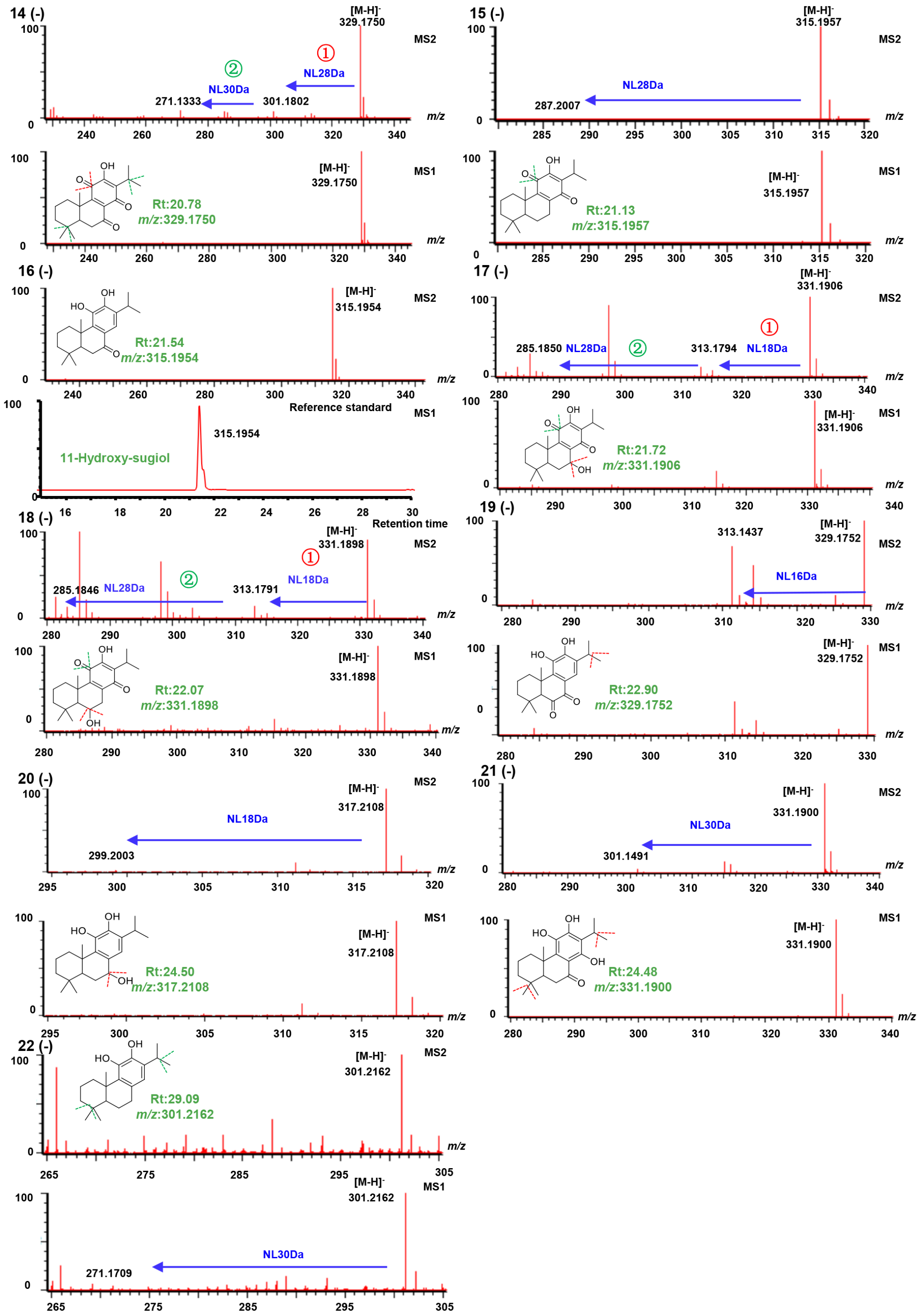


**Supplementary Figure 29. The fragmentation pattern of ATDs with modifications on rings (Group-C) in the negative-ion mode.**

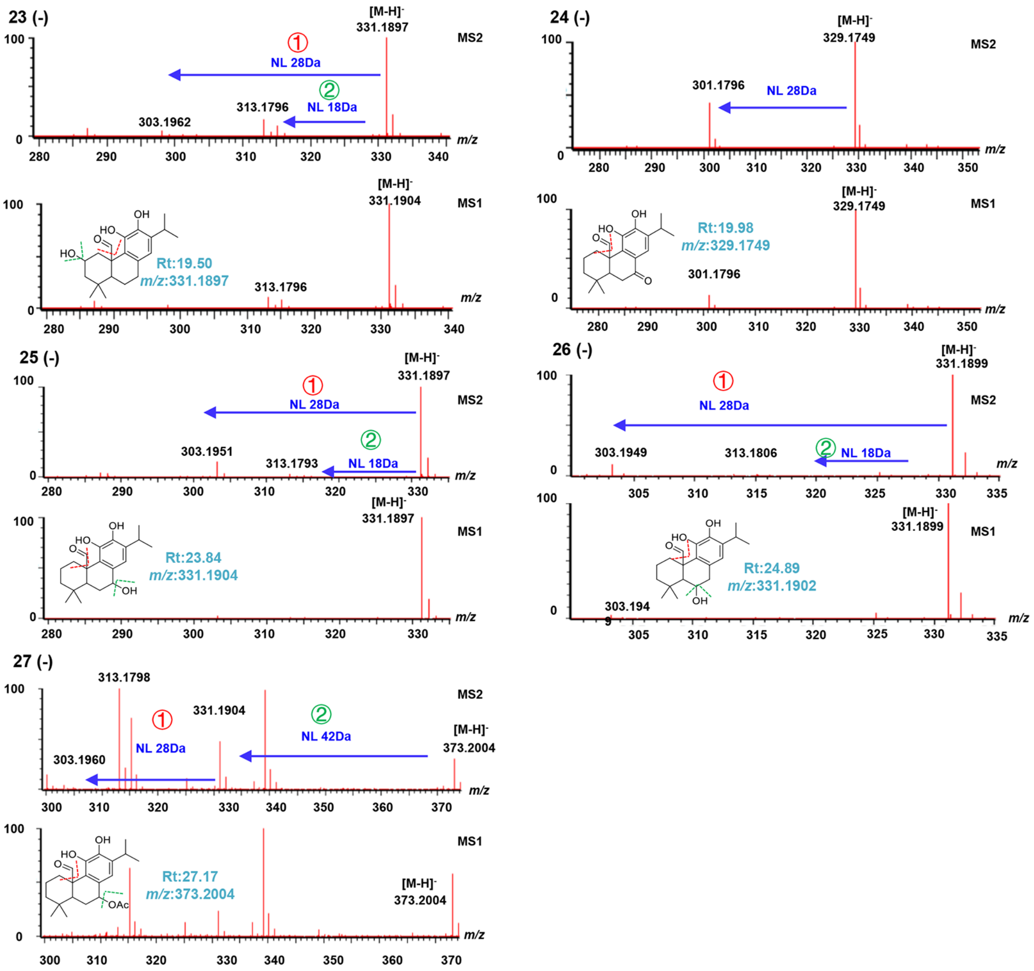


**Supplementary Figure 30. The fragmentation pattern of C-20 keto ATDs (Group-D) in the negative-ion mode.**

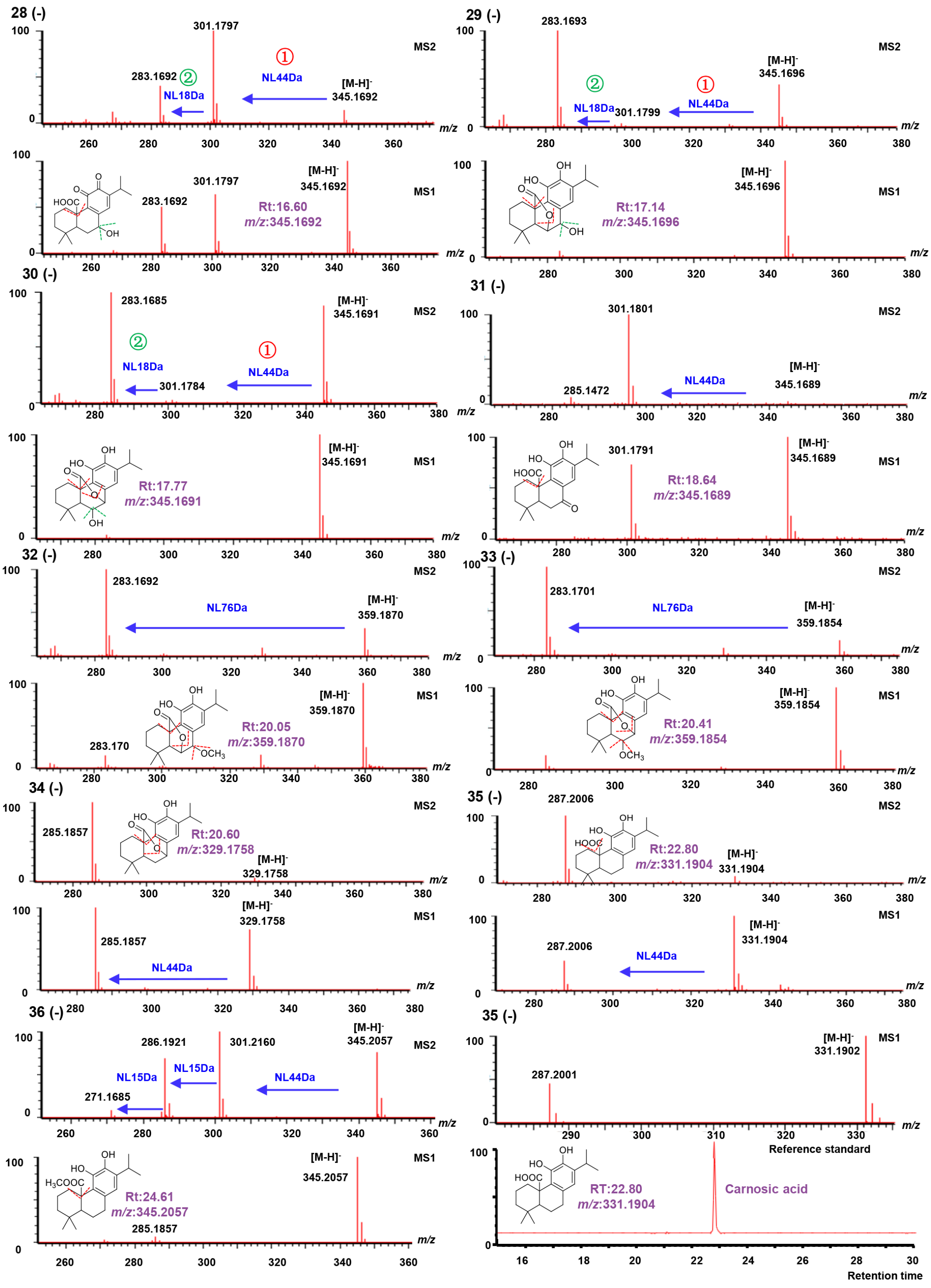

**Supplementary Figure 31. The fragmentation pattern of C-20 carboxyl and epoxyl ATDs (Group-E) in the negative-ion mode.**

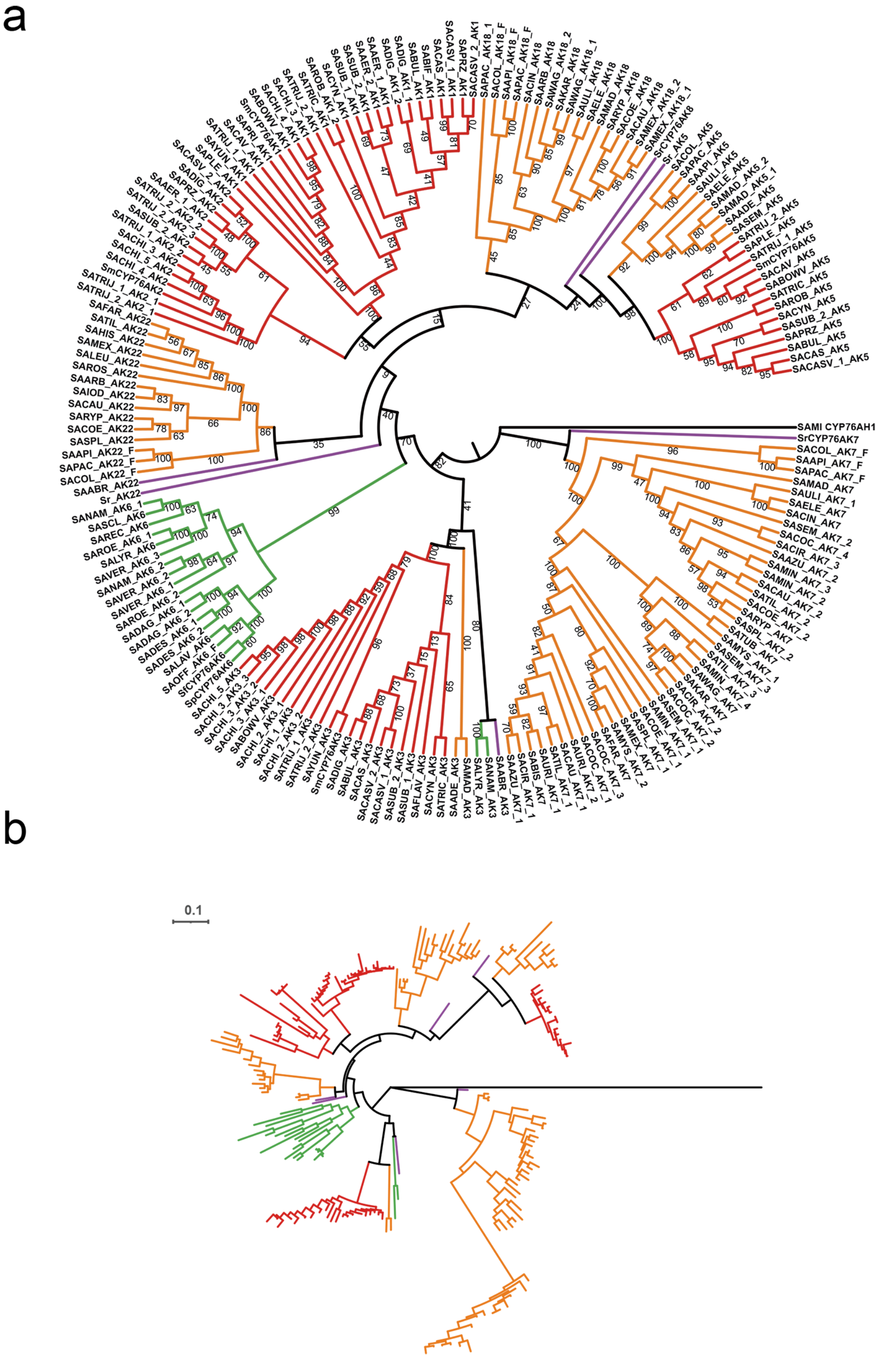


**Supplementary Figure 32. Phylogeny of the CYP76AK subfamily members**. (a) Main cladogram with gene names. Orange branches are sequences from Clade II; red are sequences from Clade IV; green are sequences from Clade I; purple are sequences from subgenera *Perovskia* and *Rosmarinus*; all other branches are black. Bootstrap values are presented near the nodes. (b) Inset shows phylogram with branch lengths. Species acronyms can be found in **Supplementary Data 1**. Source data are provided as a Source Data file.

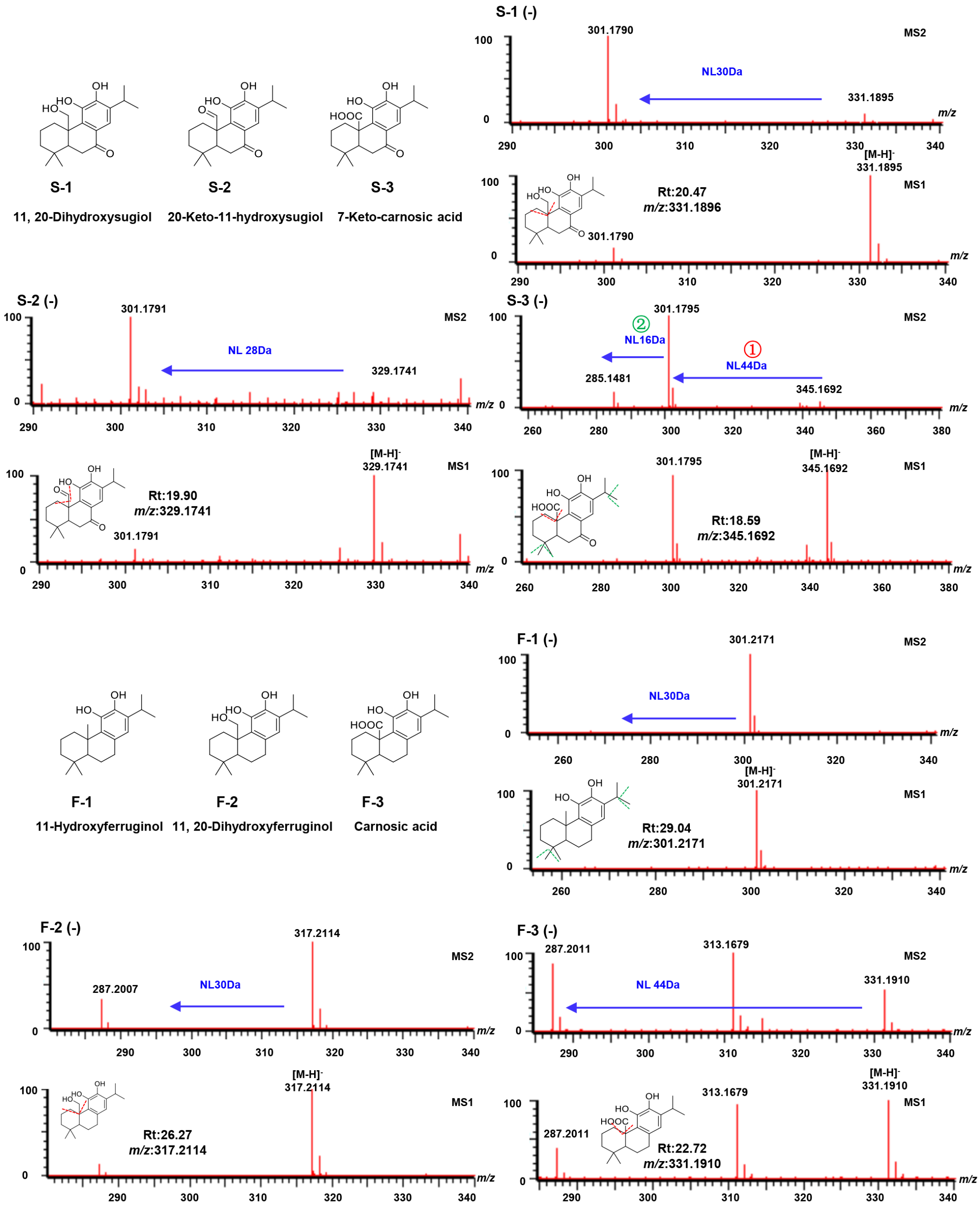


**Supplementary Figure 33. The identification and fragmentation pattern of six products (S1-3, F1-3) from *in vitro* enzyme activity assay in the negative-ion mode.**

**(a)**

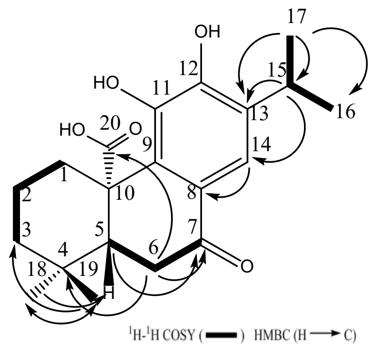
OH
HO
12
11
15
17
16
13
14
HO
20
O
9
8
1
10
2
3
4
5
6
7
O
18
19
H
$^1$H-$^1$H COSY ( ) HMBC (H → C)

**(b)**

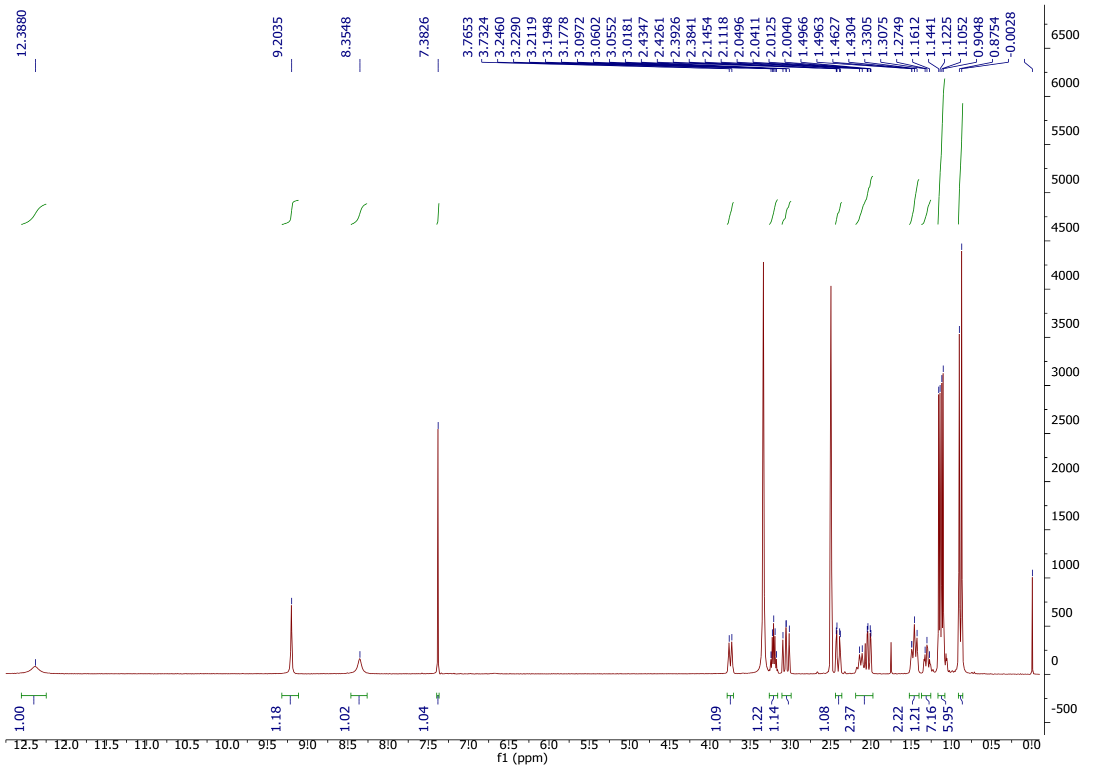
12.3880
9.2035
8.3548
7.3826
3.7653
3.7324
3.2460
3.2290
3.2119
3.1948
3.1778
3.0972
3.0602
3.0552
3.0181
2.4347
2.4261
2.3926
2.3841
2.1454
2.1118
2.0496
2.0411
2.0125
2.0040
1.4966
1.4963
1.4627
1.4304
1.3305
1.3075
1.2749
1.1612
1.1441
1.1225
1.1052
0.9048
0.8754
-0.0028
1.00
1.18
1.02
1.04
1.09
1.22
1.14
1.08
2.37
2.22
1.21
7.16
5.95
f1 (ppm)

(c)

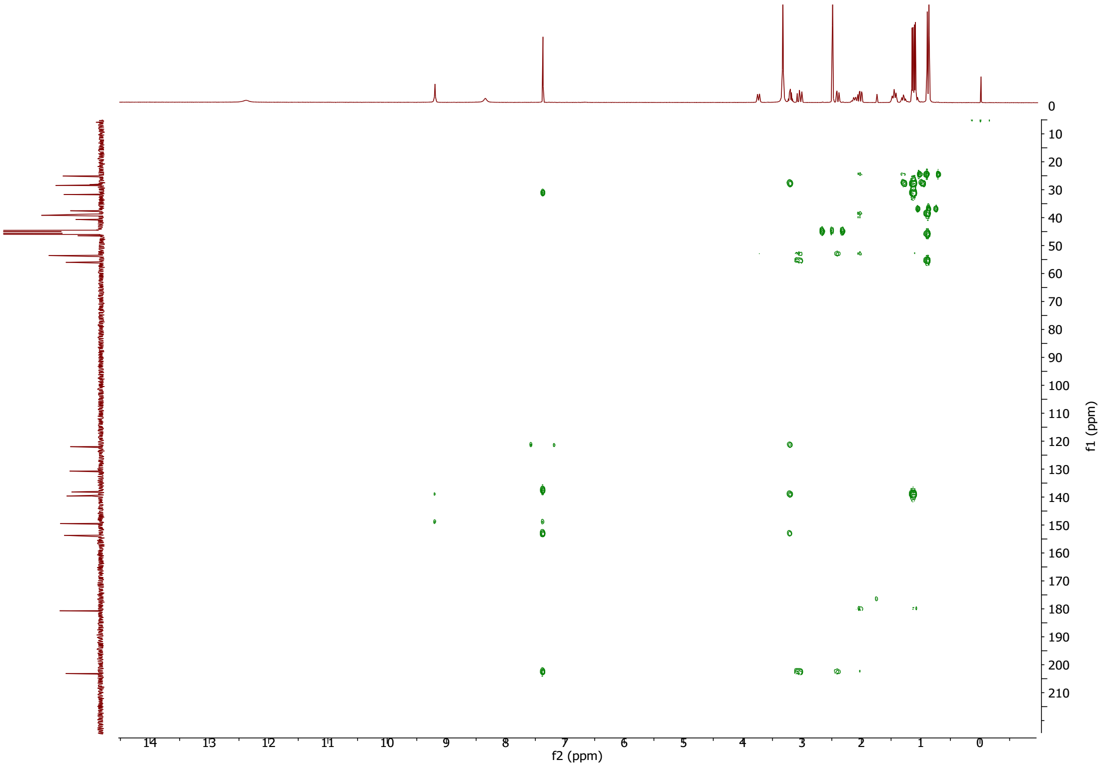


(d)

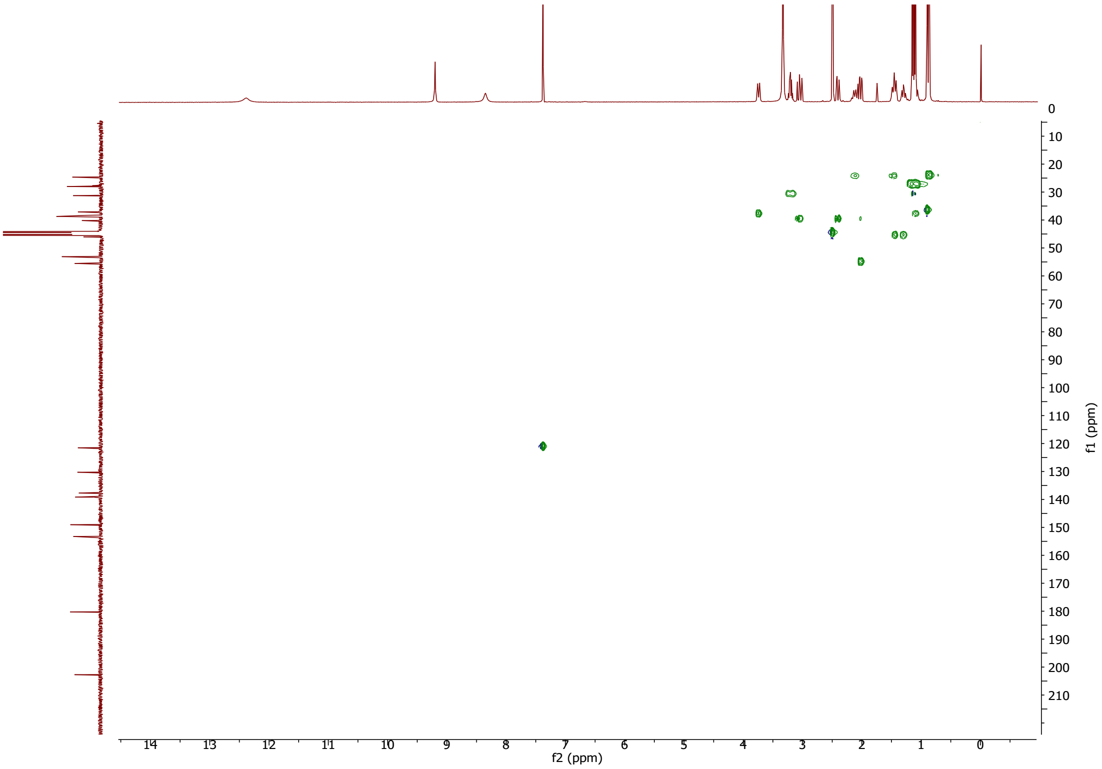

**(e)**

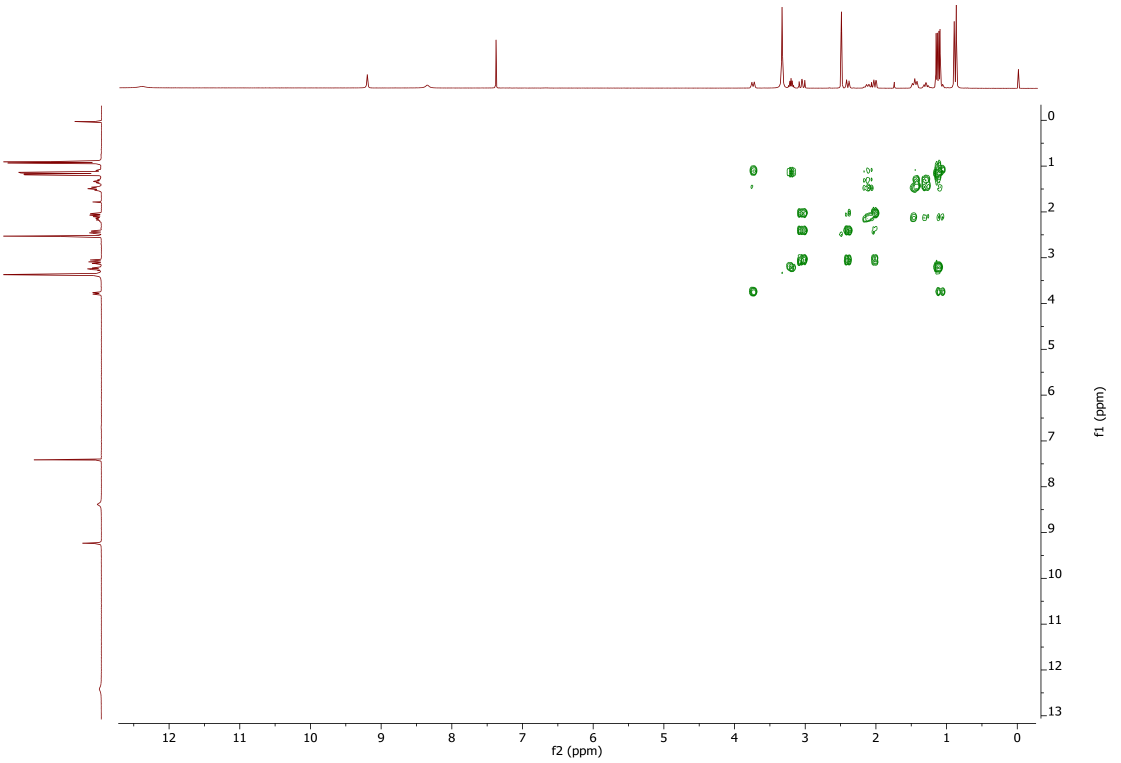


**(f)**

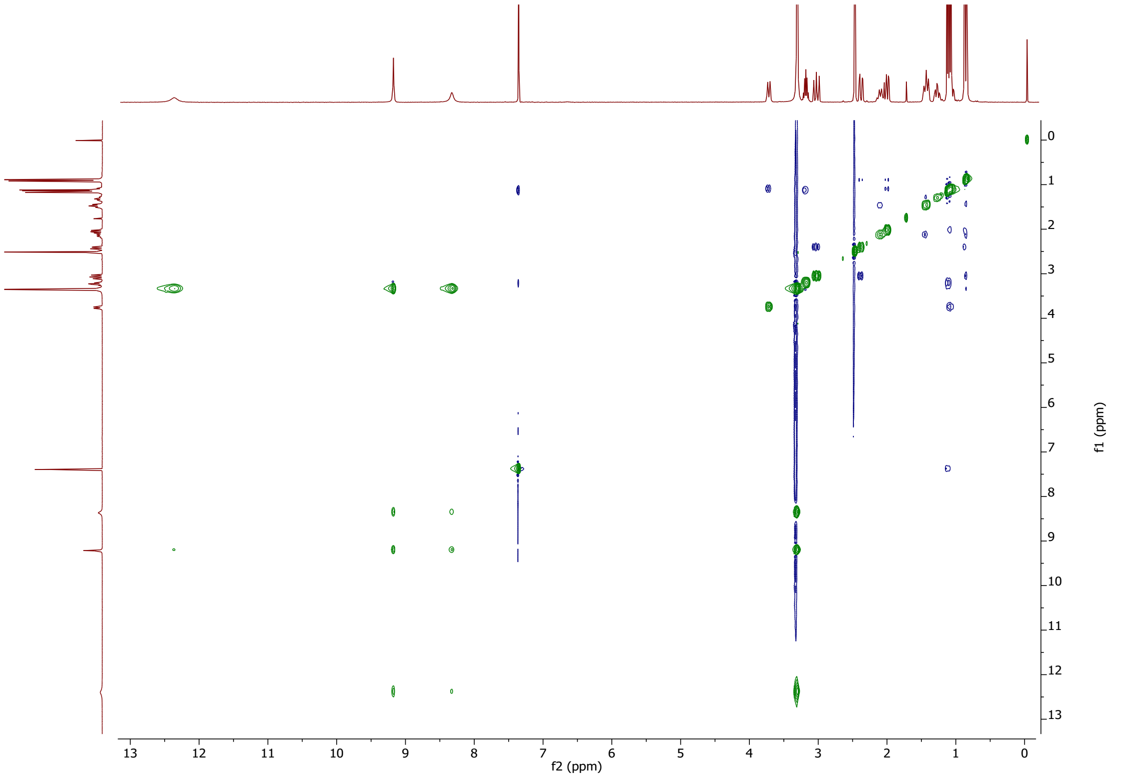

**(g)**

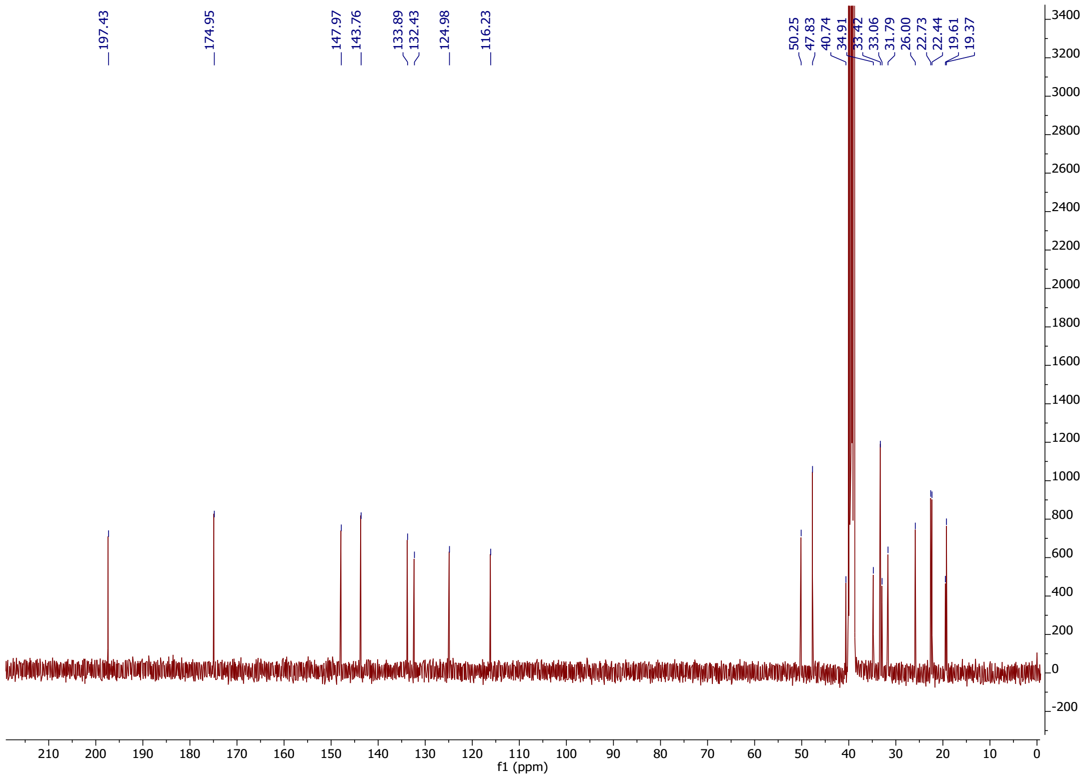


**Supplementary Figure 34. Structural elucidation of 7-keto-carnosic acid (32) by NMR.** Chemical shift assignments, based on (a) assigned HMBC and COSY correlations, (b) $^1$H NMR spectrum in DMSO-$d_6$, (c) HMBC spectrum in DMSO-$d_6$, (d) HSQC spectrum in DMSO-$d_6$, (e) $^1$H-$^1$H COSY spectrum in DMSO-$d_6$, (f) $^1$H-$^1$H NOESY spectrum in DMSO-$d_6$, (g) $^{13}$C NMR spectrum in DMSO-$d_6$.

$^1$H-NMR (400 MHz, DMSO-$d_6$) δ: 12.39 (1H, brs, 20-COOH), 9.20 (1H, s, 11-OH), 8.35 (1H, s, 12-OH), 7.38 (1H, s, H-14), 3.77 (1H, m, H-1b), 3.21 (1H, m, H-15), 3.06 (1H, dd, *J*=16.8, 14.8 Hz, H-6a), 2.41 (1H, dd, *J*=16.8, 3.2 Hz, H-5), 2.13 (1H, m, H-2a), 2.04 (1H, dd, *J*=14.8, 3.2 Hz, H-6b), 1.50 (1H, m, H-2b, overlapped), 1.45 (1H, m, H-3a, overlapped), 1.31 (1H, m, H-1b), 1.15 (3H, d, *J*=6.8 Hz, H-17), 1.11 (3H, d, *J*=6.8 Hz, H-16), 1.10 (1H, m, H-3b, overlapped), 0.91 (3H, s, H-19), 0.88 (3H, s, H-18).

$^{13}$C-NMR (400 MHz, DMSO-$d_6$) $\delta$: 197.43 (C-7), 174.95 (C-20), 147.97 (C-11), 143.76 (C-12), 133.89 (C-13), 132.43 (C-8), 124.98 (C-9), 116.23 (C-14), 50.25 (C-10), 47.83 (C-5), 40.74 (C-3), 34.91 (C-4), 33.42 (C-1), 33.06 (C-6), 31.79 (C-15), 26.00 (C-18), 22.73 (C-16), 22.44 (C-17), 19.61 (C-2), 19.37 (C-19).

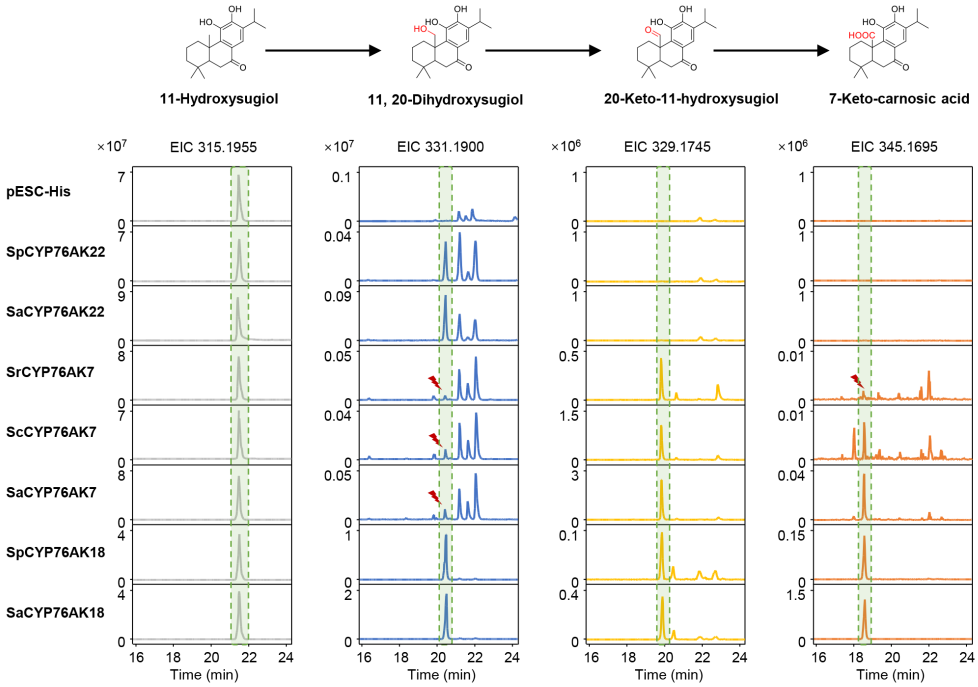


**Supplementary Figure 35. Catalytic activity of targeted CYP76AK subfamily members (CYP76AKs) with 11-hydroxysugiol.** Extracted ion chromatograms (EIC) overlay showing the in vitro catalytic activity of CYP76AKs with 11-hydroxysugiol. The sample with the empty vector (pESC-His) served as the negative control. Peaks potentially corresponding to products are labeled with the *m/z* value of 11, 20-dihydroxysugiol (*m/z* 331.1900), 20-keto-11-hydroxysugiol (*m/z* 329.1745), 7-keto-carnosic acid (*m/z* 345.1695).

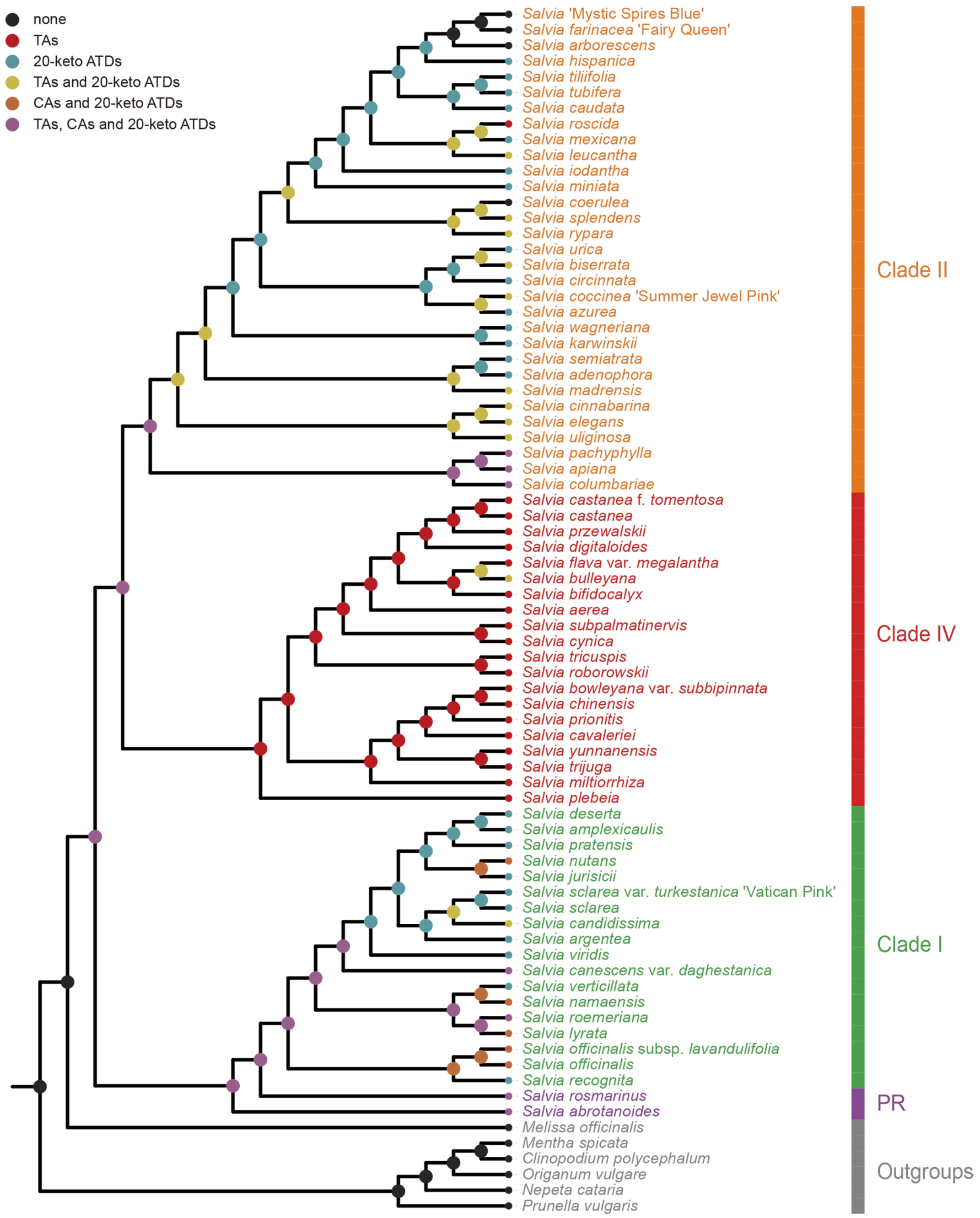


**Supplementary Figure 36. Ancestral character states of ATDs.** Genera names are listed at the right of the tree. Colored blocks delimit the major clades identified in this study. PR, subgenera *Perovskia* and *Rosmarinus*

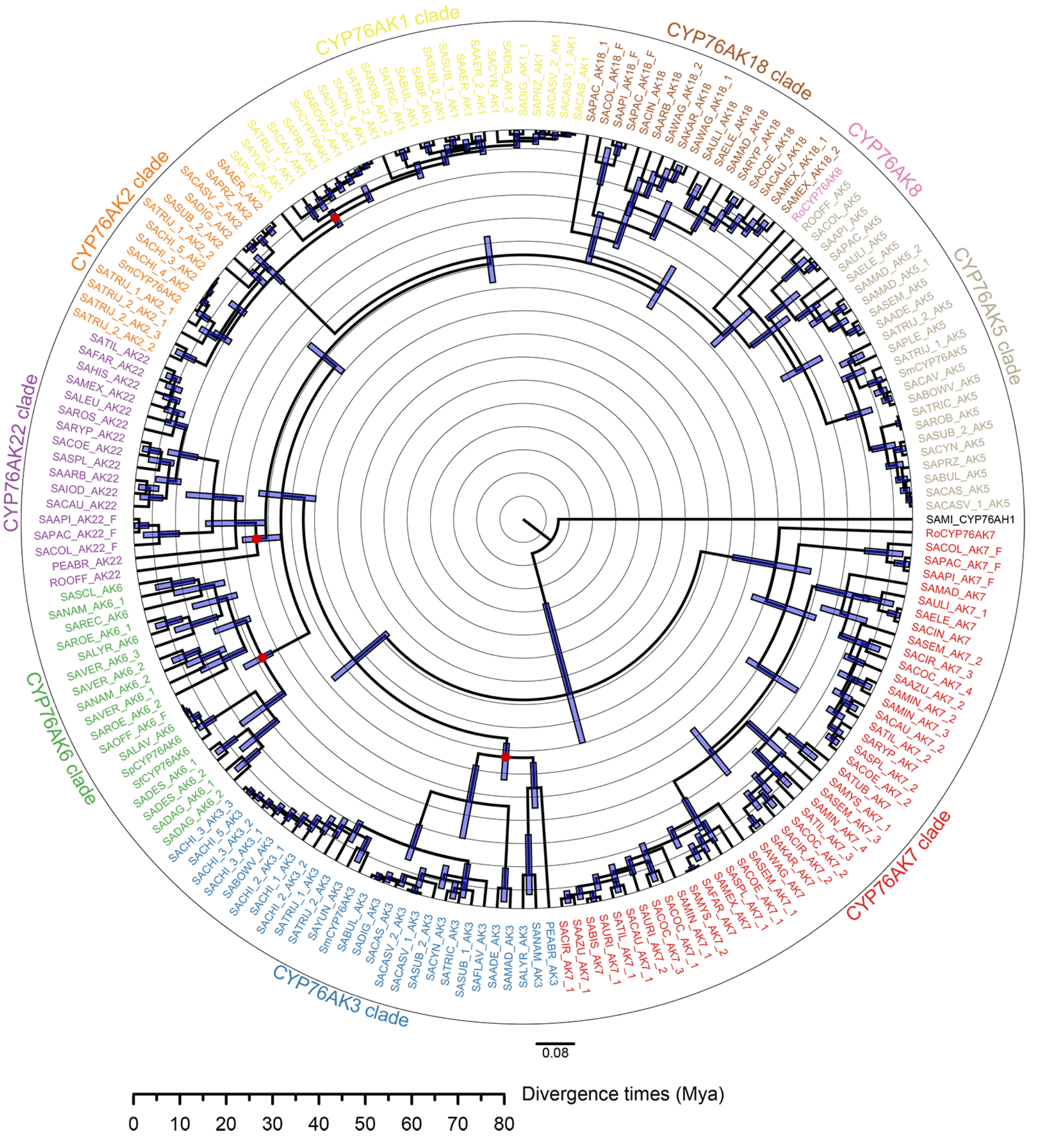


**Supplementary Figure 37.** Chronogram of CYP76AKs evolution. Divergence times (Million years ago, Mya) were estimated assuming a relaxed molecular clock, as implemented in treePL with a penalized likelihood approach. Shown here is a summary of estimated ages from 1000 dated bootstrap trees, with blue bars showing 95% confidence intervals. Red circles denote calibrated nodes. Species acronyms can be found in **Supplementary Data 1**. Source data are provided as a Source Data file.

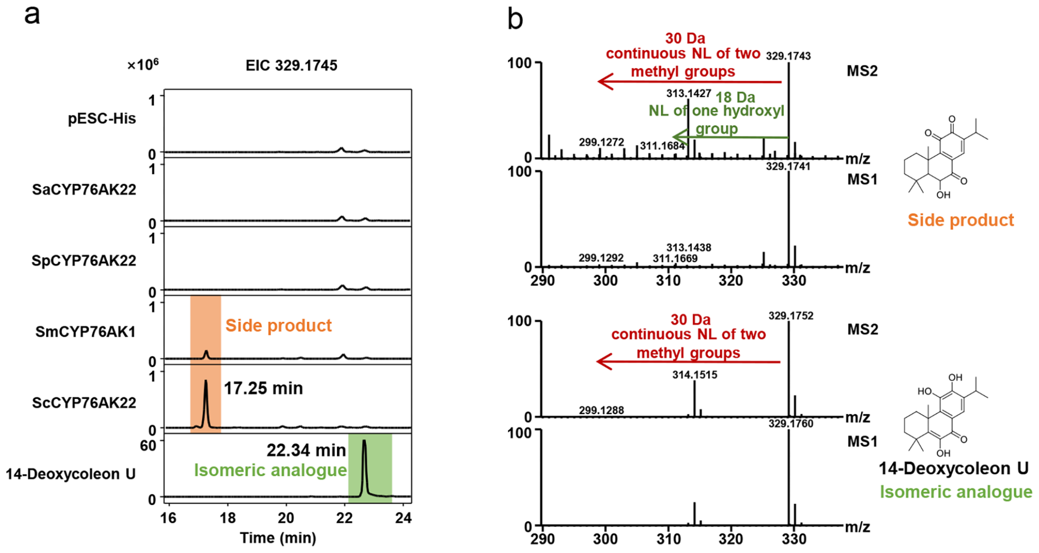


**Supplementary Figure 38. Structure elucidation of side product catalyzed by SmCYP76AK1 and ScCYP76AK22**. (a) Extracted ion chromatogram (EIC) analysis of the CYP76AK1/22 catalytic side product in vitro with 11-hydroxysugiol along with an isomeric analogue; (b) Structure elucidation of the side product based on the characteristic neutral loss and compared to the isomeric analogue.

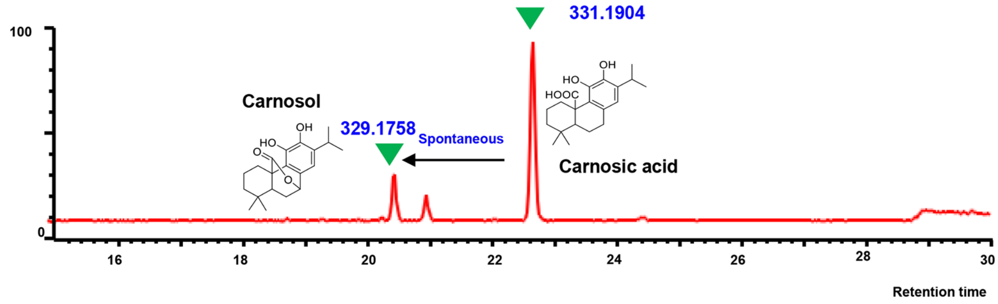


**Supplementary Figure 39. Spontaneous oxidation of carnosic acid to carnosol.**
