# Supplementary material for: Functional divergence of CYP76AKs shapes the chemodiversity of abietane-type diterpenoids in genus Salvia
Source: Nat Commun. 2023 Aug 4;14:4696. doi: 10.1038/s41467-023-40401-y (PMC10403556; doi:10.1038/s41467-023-40401-y)
Supplement: Supplementary file 3 — Description of Additional Supplementary Files [file 41467_2023_40401_MOESM3_ESM.pdf]

Description of Additional Supplementary Files:

Supplementary Data 1: List of surveyed species, including acronym, sample type, taxonomic classifications, recorded habitat, accession source, collection location, collection date, reproductive stage, number of individuals sampled, and transcriptome assembly statistics for this study.

Supplementary Data 2: Feature table of Phenolic acids fraction (1.0-18.5 min) from *Salvia* root samples in the negative-ion mode.

Supplementary Data 3: Feature table of Phenolic acids fraction (1.0-18.5 min) from *Salvia* leaf samples in the negative-ion mode.

Supplementary Data 4: Feature table of ATDs fraction (16.5-29.5 min) from *Salvia* root samples in the negative-ion mode.

Supplementary Data 5: Feature table of ATDs fraction (16.5-29.5 min) from *Salvia* root samples in the positive-ion mode.

Supplementary Data 6: Feature table of ATDs fraction (14.0-29.5 min) from *Salvia* leaf samples in the negative-ion mode.

Supplementary Data 7: Feature table of ATDs fraction (14.0-29.5 min) from *Salvia* leaf samples in the positive-ion mode.

Supplementary Data 8: Chemical Characterization of major ATDs from *Salvia* genus in both roots and leaves.

Supplementary Data 9: The number of *CYP76AKs* in all species.

Supplementary Data 10: Syntenic analysis of *CYP76AK2/3/5* genes between five Salvia genomes (*S. rosmarinus, S. miltiorrhiza, S. bowleyana, S. hispanica, and S. splendens*) with N. cataria genome as a reference outside the genus. The syntenic relationships of different genes were represented in individual tables.

Supplementary Data 11: Sequence information of all CYP76AK ancestors.

Supplementary Data 12: Primers used in this study.
